# Practice determinants for adherence to the Guide for the Comprehensive Clinical Care of Dengue Patients, Urabá (Colombia). A multifaceted approach to implementation research

**DOI:** 10.1371/journal.pntd.0012361

**Published:** 2024-08-15

**Authors:** Luisa Consuelo Rubiano, Dayana Montoya, Catalina Urrego, Santiago Alberto Morales, Paola Astrid Ríos-Tapias, Katherine Monsalve, Margarita Arboleda

**Affiliations:** 1 Instituto Colombiano de Medicina Tropical (ICMT- CES), Apartadó, Colombia; 2 Universidad CES, Facultades de Psicología, Enfermería y Medicina, Medellín, Colombia; 3 Universidad CES, Maestría en Epidemiología, Medellín, Colombia; Faculty of Science, Ain Shams University (ASU), EGYPT

## Abstract

**Introduction:**

Dengue is a significant public health issue in the Urabá region, accounting for 37.5% of morbidity and 41.7% of mortality resulting from dengue in the department of Antioquia (Colombia) in 2018. Clinical Practice Guidelines (CPGs) are tools based on Evidence-Based Medicine, intended for medical personnel to bridge the gap between proven intervention efficacy and clinical decision-making. This study aims to identify barriers and facilitators in the implementation of CPGs for dengue patient care by healthcare officials in the municipalities of the banana axis in the Urabá region.

**Methodology:**

From a multifaceted approach to implementation research, a mixed method study that combines qualitative and quantitative approaches, was conducted during the years 2020 and 2021, using combined instruments to identify determinants (Guide Indicative Factors, Individual factors of health professionals, Patient factors, Professional interactions, Incentives and resources, Capacity for organizational change, and lastly Social, political, and legal factors) affecting adherence to the Comprehensive Clinical Care Guide for Patients with Dengue (GACIPD). Semi-structured interviews and focus groups with healthcare workers were conducted to assess determinants based on clinical experience. Questionnaires on determinants of GACIPD adherence, using an adapted version of the Chronic Disease Implementation Checklist (TICD), were also employed. Qualitative analysis of the interviews and focus groups used a concept-based coding framework. The questionnaire responses were analyzed using Likert scaling and frequency counts of determinants within and across domains. Participants included general practitioners, other health professionals, researchers, academics, and administrators.

**Results:**

There was a total of 103 participants in focus groups, 7 in semi-structured interviews, and 136 participants through questionnaires. Among the domains studied, the identification of barriers and facilitators emphasized institutional factors and individual factors. Organizational change capacity was identified as a major barrier, with only 3.6% of respondents indicating that their institution adjusted the prioritization of adequate care according to the guideline. The GACIPD domain received the highest facilitator rating, with 66.7% acceptance due to its practicality, simplicity, clarity, documentation, and ease of implementation, despite this, only 10% of professionals completely agree that their work is in accordance with the GACIPD. The determinant of patient factors was significant in the negative perception of adherence to GACIPD.

**Conclusions:**

Although barriers outweighed facilitators for GACIPD adherence, determinants for its use were generally positive, as most participants reported it as being a complete, documented, and easy-to-implement guide. The lack of knowledge of the guidelines impacting health professional’s decision making was identified as a potentially modifiable barrier, and educational strategies could be implemented to overcome it. The region requires greater emphasis on the management of chronic health conditions, comorbidities, and coinfections of dengue with other endemic diseases.

## Author summary

Dengue is a significant public health issue in the Urabá, this region reported around 40% of morbidity and mortality for the disease in the department of Antioquia (Colombia) in 2018. Clinical Practice Guidelines are tools intended for medical personnel to bridge the gap between proven intervention efficacy and clinical decision-making. A study was conducted s to identify determinants affecting adherence to the Comprehensive Clinical Care Guide for Patients with Dengue. Focus groups, questionnaires, and interviews were used. Participants included general practitioners, other health professionals, researchers, academics, and administrators. The barrier most frequently identified by participants was organizational change capacity, however individual health professionals also identified a lack of interest in self-learning and knowledge of the guidelines. On the other hand, among facilitators, they identified the guideline’s inherent characteristics. Although barriers outweighed facilitators for GACIPD adherence, determinants for its use were generally positive, as most participants reported it as being a complete, documented, and easy-to-implement guide. The lack of interest in self-learning and knowledge of the guidelines was identified as a potentially modifiable barrier and educational strategies could be implemented to overcome it.

## Introduction

The resurgence of dengue worldwide has a direct impact on the health of people in endemic countries. The World Health Organization (WHO) issued epidemiological alerts for dengue in the Americas region on November 21, 2018 [1] and again in February 2019, reporting 560,586 cases of dengue and 336 deaths in the region between epidemiological weeks 1 and 52 of 2018 [2]. In Colombia, the National Institute of Health (NIH) issued an epidemiological alert for dengue on February 12, 2019, and a contingency plan was implemented to combat the national epidemic [3].

Dengue poses a significant public health problem in the Urabá region. The disease burden there is attested by its contribution of 37.5% (1426/3805) of the total reported cases in the department of Antioquia in 2018. This represents 3.5 times the incidence rate (205.5 vs 58.3 cases/100,000 inhabitants) and 41.7% (5/12) of the department’s mortality resulting from dengue, highlighting the magnitude of the problem in the Urabá region [4].

After analyzing fatal cases of dengue fever in Urabá, failures were identified at 2 levels. The first is that of the community level and relates to delays in recognizing warning signs by caregivers and/or family members. The second is that of the healthcare level, including the number of consultations prior to hospitalization (up to 7 consultations) and the clinical recognition and management of critical states (compensated shock, irreversible shock) by medical personnel in the emergency services of primary and secondary healthcare institutions. The latter is particularly salient in the municipalities of Turbo and Apartadó.

Considering these aspects that point to failures in healthcare, it is important to emphasize the development of guidelines for the care of patients with dengue fever. These guidelines are intended to be practical tools for healthcare personnel, including physicians, residents, nurses, medical students, microbiologists, epidemiologists, and health unit managers. This is clearly stated in the introduction of The Pan American Health Organization’s guides: Dengue: guidelines for patient care in the Region of the Americas, Second Edition, 2016 [5]. They are intended to ensure more timely and accurate treatment of dengue fever cases, from primary healthcare to specialized units at the secondary and tertiary levels [6].

Clinical Practice Guidelines (CPGs) are tools that adhere to Evidence-Based Medicine (EBM) principles. They are designed for medical personnel to bridge the gap between proven efficacy and clinical decision-making [7–9]. EBM is a strategy to standardize variability, improve quality, and reduce errors and costs in the prevention, diagnosis, and treatment of specific diseases [7–10].

Various instruments, including protocols, procedures, algorithms, and in Colombia the GACIPD [11–14], are available to guide clinical decisions. There are also institutions, including the Ministry of Health and Social Protection (MSyPS), the National Institute of Health (NIH), and the Institute for the Evaluation of Health Technologies and Research (IETSI), whose roles involve promoting and disseminating the appropriate use of these tools within the National Health System. However, adherence of healthcare personnel to these recommendations is limited.

The existing gaps in evaluating the adoption and adaptation of guidelines at different levels of care require an analysis of the context for both the populations and healthcare workers. It is also necessary to identify bottlenecks at the individual, group, and organizational levels, necessitating the study of factors that affect CPG adherence. The changing models of care in patient pathways, the updating of guidelines [11,15,16], and the structure of medical care pose significant challenges to CPG implementation.

The objective of this study is to describe the determinants of GACIPD implementation for dengue care and to identify barriers and facilitators that influence its use in primary health centers and general hospitals at the secondary level in four municipalities of the banana axis. This qualitative and quantitative approach aims to improve our understanding of both healthcare workers’ perspectives and the identification of factors crucial to strategies enhancing its effectiveness. All those involved in the care of dengue patients were included, not only doctors, but also microbiologists, nurses, quality control supervisors, and decision makers.

## Methodology

### Ethics statement

Data were anonymized to maintain the confidentiality of the analysis. All participants gave written, informed consent to participate in the study. The study was conducted in accordance with the guidelines established in the Declaration of Helsinki and Colombian Decree 8390 of 1993. It was approved by the Bioethics Committee of the Colombian Institute of Tropical Medicine, through Act Number 66 of 14-06-2019.

Considering the importance of context in the implementation of care guidelines, this methodology allows information to be obtained, not only from direct users, but also from all those who affect the successful execution of the same, in logistical, financial, and administrative aspects.

**Fig 1:**
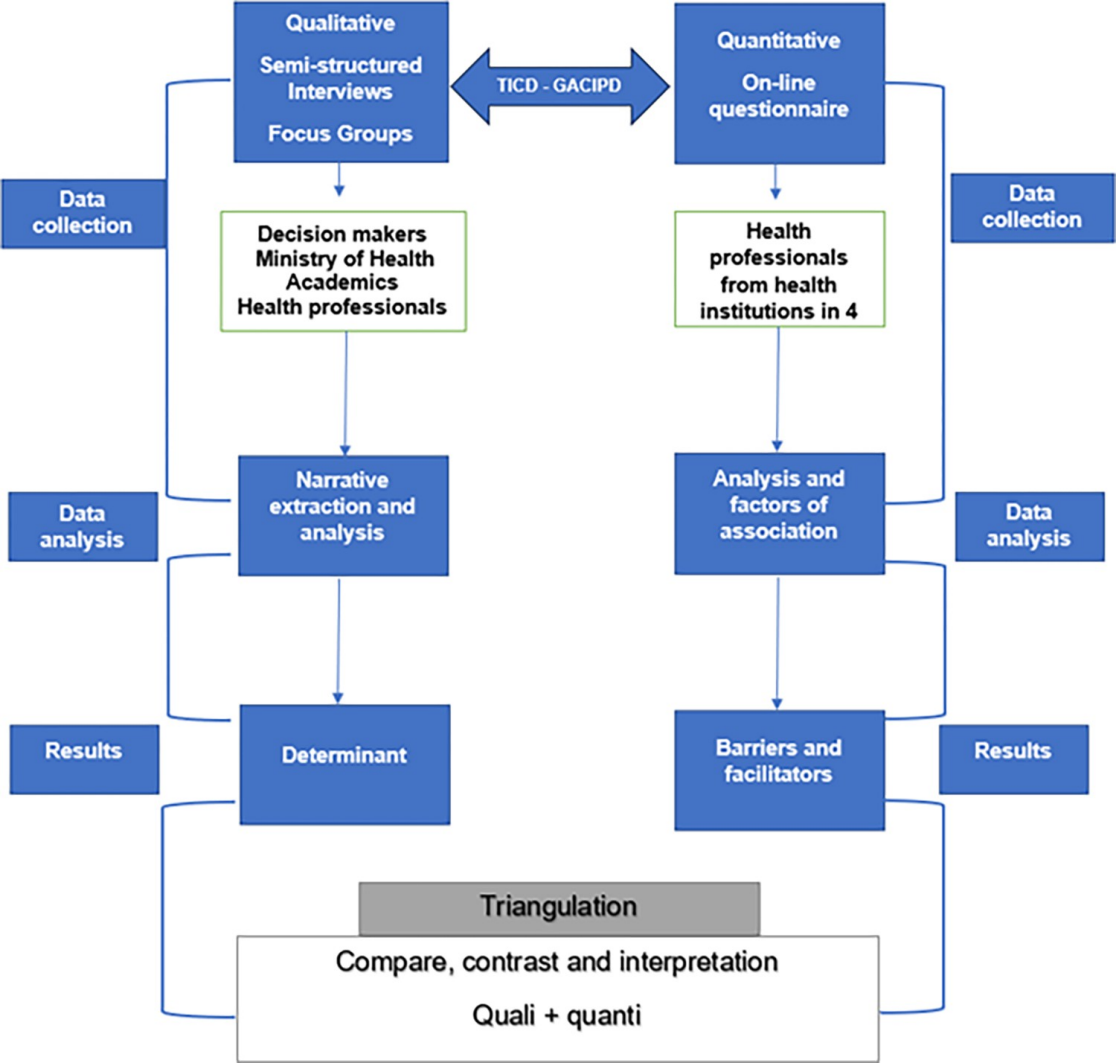
Convergent mixed-methods design using parallel data collection [17].

From a multifaceted approach to implementation research, a mixed-method study that combines qualitative and quantitative approaches, using combined instruments (focus groups, key informant interviews, semi-structured surveys, and online questionnaires) (Table A in S1 Appendix), with a convergent triangulation design (Fig 1) [17,18], the determinants of clinical practice were identified and the barriers and facilitators for adherence to the GACIPD were described; This design facilitated the analysis of the data separately, allowing comparisons of the results at the time of interpretation and discussion, which can be seen in the statistical results of the surveys, followed by the categorical information that emerged from the focus groups and interviews, which led to corroborating the quantitative data. The Consolidated Criteria for Reporting Qualitative Research (COREQ) checklist [19] (Table A in S2 Appendix) and the STROBE checklist for observational studies [20] (Table B in S2 Appendix) guided data collection and analysis, along with Flottorp’s comprehensive checklist, the TICD checklist [21,22], to identify the following determinants: Guide Indicative Factors, Individual factors of health professionals, Patient factors, Professional interactions, Incentives and resources, Incentives and resources, and lastly Social, political, and legal factors.

The activities were carried out in the Urabá region, in the municipalities known as the banana axis (Turbo, Apartadó, Carepa, and Chigorodó) during the years 2020 and 2021. There was a total of 246 participants among healthcare professionals from the first and second-level health institutions, as well as decision-makers from government and academic spheres across the country. A background diversity in the healthcare professionals was selected intentionally to identify better barriers and facilitators for CPG implementation at different levels, from direct patient care to institutional management, and policy making. Due to the conditions imposed by the Covid-19 pandemic, data collection was carried out both virtually and in person.

### Study population

The activities were carried out in the Urabá region, in the municipalities known as the banana axis (Turbo, Apartadó, Carepa, and Chigorodó) during the years 2020 and 2021. There was a total of 246 participation among healthcare professionals from the first and second level health institutions.

The study defined healthcare professionals as those professionals within healthcare institutions with direct or indirect responsibilities in patient care, this included: doctors, professional nurses, nursing technicians in charge of direct patient care; microbiologists in charge of laboratory work; quality control supervisors, and auditors in charge of reviewing that patient care was adequate and give feedback to other professionals; and administrators who determine institutional policies. There were 103 through focus groups (Table 1), seven decision-makers from the government (Ministries of Health) and academic (Deans of faculties of health sciences) institutions from different parts of the country, through semi-structured interviews (Table 2), and 136 participants through surveys (Table 3). Due to the conditions imposed by the Covid-19 pandemic, data collection was carried out both virtually and in person.

**Table 1:** Characteristics of focus group participants. n = 103.

Focus groups		n	%
Institutions	Clinics	5	50.0
	Hospitals	3	30.0
	Health centers	2	20.0
Participants*	Physicians	41	39.8
	Nurses	14	13.6
	Nursing technicians	33	32.0
	Microbiologist	11	10.7
	Quality/Public Health	4	3.9
Sex	Female	70	68.0
	Male	33	32.0
Years of experience	1 or less	9	10.0
	2–4 years	10	11.0
	5–9 years	26	27.0
	10 or more	58	59.0
Modality	On-Site	8	80.0
	Tele-assisted	2	20.0

*Physicians, Nurses, and Nursing Technicians with a role in direct patient care of dengue patients. Microbiologists, responsible for processing clinical samples from dengue patients. Quality/Public Health corresponds to administrative workers in charge of reviewing records from patient care and upholding adherence to guidelines by other professionals.

**Table 2:** Characteristics of the participants in the semi-structured interviews. n = 7.

Semi-structured interview		n	%
**Institutions**	Ministry of Health	2	28.6
	Academic	5	71.4
**Sex**	Female	4	57.1
	Male	3	42.9
**Years of experience**	5 o 9 years	4	57.1
	10 or more	3	42.9

**Table 3:** Questionnaire participant characteristics.

Variable	Categories	N = 136	%
Sex	Female	102	75.0
	Male	34	25.0
Profession*	Nursing technicians	68	50.0
	Physicians	33	24.3
	Nurse	21	15.4
	Microbiologist	6	4.4
	Administrative	4	2.9
	Pediatrician	1	0.7
	Internist	1	0.7
	Epidemiologists	2	1.4
Municipio	Apartadó	95	69.9
	Chigorodó	17	12.5
	Carepa	14	10.3
	Turbo	10	7.3
Years of experience	1 or less	18	13.2
	2–4 years	39	28.7
	5–9 years	23	16.9
	10 or more	56	41.2

*Nursing Technicians, Physicians, Nurses, a pediatrician, and an internal medicine specialist with a role in direct patient care of dengue patients. Microbiologists, responsible for processing clinical samples from dengue patients. Epidemiologists and Administrative workers with functions, reviewing records from patient care and upholding adherence to guidelines by other professionals.

Samples for each institution were represented in an equivalent proportion of the total professionals with the same position in the institution. The only definition of which professionals to approach was their position within the institution and their availability during the visit to the institution.

### Semi-structured interviews

A questionnaire composed of 14 questions based on the 7 domains (Guide Indicative Factors, Individual factors of health professionals, Patient factors, Professional interactions, Incentives and resources, Capacity for organizational change, and lastly Social, political, and legal factors) proposed by Flottorp [21] was designed (Table A in S1 Appendix). Decision-makers involved in vector-borne diseases, including those in legislative and regulatory positions, as well as academic professionals from various health science faculties, were invited to participate. The interviews were conducted virtually by a member of the research team specialized in qualitative research. The research team was present during the interviews but did not intervene. Each interview lasted approximately 60 minutes, as consented to by the interviewees. The collected data were treated as confidential and were recorded and transcribed.

The semi-structured questionnaire focused on the question: "What are the barriers and facilitators to GACIPD adherence for the care of the dengue-affected population?". The interviews were exclusively directed at decision-makers in the educational and regulatory sphere to assess adaptation in the educational and managerial context for guiding policies in healthcare worker training. For this reason, interviews were not conducted with healthcare workers in executive roles to evaluate the adoption of the guidelines. Convenience sampling was used where, during the interviews, the comprehensive Flottorp checklist, which categorizes determinants of practice into seven domains, was employed (Table A in S1 Appendix).

The interview questionnaire underwent a validation process through a pilot test conducted with volunteer personnel from the study area and specialists in vector-borne diseases. This test assessed the completeness, relevance, applicability, simplicity, logic, clarity, usability, propriety, and utility of each question in each domain, following the recommendations in the TICD checklist [21] (Validation of semi-structured interview questions/structured questionnaire for health personal in S1 Appendix). After discussing the results with the research team, the questionnaire was adjusted accordingly.

The questionnaire and GACIPD were sent to decision makers before the semi-structured interviews, because with them the study wanted to get information about policies in their respective institutions, not the interviewee’s clinical knowledge. Despite the risk of bias, given the difficulties of accessing a head member of government or academic institutions, prior knowledge of the questions allowed for faster and more fluid interviews, prepared answers also allowed them to verify standing practices in their institutions.

### Focus group discussion

Professionals from health centers, hospitals, and clinics in the municipalities of the banana axis were invited to participate using convenience sampling. The invitation was extended to pediatricians, internists, general practitioners, nurses, microbiologists, nursing technicians, epidemiologists, auditors, and administrators from different institutions involved in clinical and public healthcare. To ensure a diverse range of opinions, managers and medical coordinators of each institution were requested to invite personnel with different responsibilities in patient care.

The focus groups had an approximate duration of 1.5 hours and continued until data saturation was reached. During the discussions, the semi-structured interview questionnaire addressing all domains was used (Semi-structured Interview in S1 Appendix) [21]. No prior relationship was established between the moderator and the participants. The sessions were moderated by SM, a researcher experienced in qualitative research, and MA, a physician specializing in tropical diseases. Members of the research group also participated as observers. Eight in-person focus groups were conducted, while two were held virtually due to the Covid-19 health emergency.

### On-line questionnaires

An inventory of all healthcare workers in the mentioned municipalities was created, with authorization from the corresponding institutions. Contact information for each individual was obtained, and they were invited to participate in the online questionnaire (Online questionnaire in S1 Appendix). Initially, the questionnaire was conducted online, but due to a poor initial response, a portion of it was carried out in person. This was done through a direct visit by the researchers to each institution, during which available healthcare professionals were approached, and asked if they had filled in the online questionnaire, if the answer was no, they would be invited to take part in the study, if agreed a physical questionnaire was given to them to answer on their own and turn in to the researchers once completed. Intentional sampling was used to ensure representation from all institutions and types of professionals. This questionnaire had an average duration of 30 minutes.

The questionnaire followed the same design guidelines as the interview and focus groups, based on the TICD checklist [21]. Participation was developed virtually, through a Google Forms format based on the Likert scale. The validation process was carried out in a virtual pilot test conducted with volunteer personnel from an area other than the one under study. This test evaluated the completeness, relevance, applicability, simplicity, logic, clarity, usability, suitability, and utility of each of the questions in each domain, the strengths and weaknesses of the questions in the questionnaire, and the likelihood of it being carried out.

Given that participation in the study was not obligatory, there is a voluntary response bias that could not be controlled, however, samples for each institution were represented in an equivalent proportion of the total professionals with the same position in the institution. The only definition of which professionals to approach was their position within the institution and their availability during the visit to the institution. The information gathered both online and in person was analyzed through the total number of responses, it was anonymous, and individual questionnaires were not analyzed independently to ensure confidentiality.

### Sample size

"With a population of 900 healthcare workers involved in the dengue guideline adherence process in the banana-growing region municipalities, a sample size of 140 individuals was calculated using OPENEPI. This sample was assigned with an 80% confidence level, a 5% margin of error, and a prevalence of 50%.

A total of 136 questionnaires were obtained and distributed proportionally based on the number of healthcare workers from each profession and each participating institution. The selection of individuals who completed the survey was done randomly, with prior informed consent from each participant.

### Data analysis

The interviews and focus groups were transcribed and analyzed by independent researchers. The data were initially coded to fit the domains of the adapted TICD checklist, taking into account the context of healthcare in the Urabá region. Next, the data were coded deductively based on the adapted TICD determinants (Table A in S3 Appendix). Coding was compared between researchers, and any conflicts were discussed and resolved with the assistance of a third researcher. The data analysis was based on the textual transcription of the information and its subsequent reading, which allowed us to organize it into a content matrix designed in Word 2016 based on the predefined categories. This process involved identifying recurrences, divergences, and revealing emerging categories. From there, thematic matrices were designed to fill each of them with content from the generated information. Subsequently, subcategories were created, starting from the most recurrent to the least frequent, thus achieving a comprehensive and refined view of the information.

Regarding the questionnaire data, an analysis was performed for each domain by examining the frequency count of how determinants were distributed within each category. A mean analysis was conducted per domain, stratified into low, medium, and high, considering the minimum and maximum scores obtained in each domain based on the number of categories. For each set of categories, the mean, standard deviation, and Cronbach’s alpha of the total questionnaire were calculated. We used Kruskal-Wallis (for nonparametric data) to analyze continuous data such as Likert scale scores from questionnaires, comparing by profession and years of experience. Categorical data were presented with the number and percentage of participants, and continuous data as mean and standard deviation. Statistical significance was defined by p ≤ 0.05.

The analysis of the interviews and focus groups was carried out by a researcher with expertise in qualitative research, while the questionnaire data were analyzed by the epidemiologists of the research group using Excel.

In the framework of research implementation, the selection of a significant number of stakeholders offers the opportunity to identify the acceptability and appropriation of individuals and the institution. This is because the guidelines need to be known by all parties involved and determine individual and collective responsibilities regarding dengue case management. That’s why all the actors involved in the care and quality assurance of care in each institution were engaged. Using various tools allowed for the participation of a wide range of actors, different perspectives, and contexts.

## Results

### Interview and focus group results

The results of the focus groups and semi-structured interviews, along with the characteristics of the participants, are presented in Tables 1 and 2 respectively. The results are organized by domain and determinant and they include representative quotes from the participants (Table B in S4 Appendix). Relevant themes were identified from the responses for each determinant based on the TICD framework. Barriers and facilitators were identified within each determinant.

### Questionnaire results

A total of 136 professionals completed the questionnaire. The characteristics of the respondents are presented in Table 3. Seventy-five percent of the respondents were female, with nursing technicians comprising half of the participants. Physicians accounted for 24.2% of the respondents and 41.2% of the participants had more than 10 years of experience.

A rating scale or scoring system was developed for the Likert scale questionnaire based on the data distribution for each of the domains. The questionnaire’s validity was assessed using Cronbach’s alpha, which yielded a high score of 0.94, indicating good instrument performance (Table 4).

**Table 4:** Statistical analysis of the perception of the determinants of GACIPD adherence. Urabá 2021 Online Questionnaire (n = 136).

Average	151.4
Scale	44–103 LOW 104–162 MEDIUM 163–220 HIGH
Median	157
Mode	157
Standard deviation	31.4
Variance	985.03
Cronbach’s alpha	0.94

### Determinants of GACIPD adherence

In the domain of **Determinants of the guide**, a significant percentage of participants (between 74.3% and 88.2%) expressed support for the content of the GACIPD. They believed that following the guideline improves the quality of care, enhances the clinical outcomes of patients, offers advantages for professional practice within their institutions, and indicates knowledge of the guideline content (Domain A in S5 Appendix).

Regarding **professional responsibilities**, between 69.8% and 83.1% identified that following the recommended procedures, actions, or activities outlined in the GACIPD is part of their job. They also reported that their institutions provide the necessary support (such as leadership, resources, assistance, and time) to use the guideline effectively. Moreover, they found the recommended procedures easy to incorporate into their practice and considered it easy to find information on dengue within the GACIPD (Domain B in S5 Appendix).

Regarding **Individual factors of health professionals**, approximately 52.9% believed they were familiar with the guidelines for comprehensive clinical care of patients with dengue. Around 55.1% agreed that their knowledge or experience with the guidelines affects their adherence to the comprehensive care pathway for dengue (RIAS). Less than 60% of professionals reported that their work aligns with the GACIPD, they actively adopt the guideline, and the continuing education they have received on the GACIPD has been effective (Domain B in S5 Appendix).

In terms of **patient factors**, only 27.2% of the professionals considered the level of patient knowledge about the recommendations included in the GACIPD to be adequate. However, 65.4% agreed that patients’ beliefs or knowledge influence medical care, and 77.9% agreed that medical care enables patients to adhere to the recommendations in the guideline (Domain C in S5 Appendix).

Regarding **Professional interactions,** 86.8% agreed that the guideline requires the interaction of health teams. However, only 53.7% agreed that there is effective referral and counter-referral of patients between levels of care, following the recommendations of the GACIPD (Domain D in S5 Appendix).

Regarding **Incentives and resources**, 83.1% of professionals supported the availability of resources to ensure adherence to the GACIPD (financial, human, structural, or supplies). However, only 23.5% were in favor of the institutional existence of incentives for health professionals to adhere to the guideline. Furthermore, 62.5% agreed that the institutional information system facilitates adherence to the guideline (Domain E in S5 Appendix).

Regarding the **Capacity for organizational change,** between 60.3% and 69.9% stated that organizational changes are necessary to ensure adherence to the guideline. They also expressed that institution directors have the power to implement changes to improve adherence. Additionally, they were in favor of institutional guidelines or policies that facilitate the necessary changes for its adoption. However, only 47.9% believed that the institution adjusts the prioritization of adequate care in accordance with the guideline (Domain F in S5 Appendix).

In terms of **Social, political, and legal factors,** 67.6% of participants agreed that institutional policies facilitate adherence to the GACIPD guideline. Additionally, 34.6% agreed that the economic conditions of their institution affect adherence to the guideline, and 62.5% agreed that the type of contract under which they worked with their institution facilitates adherence to the GACIPD (Domain G in S5 Appendix).

### Bivariate analysis

The bivariate analysis revealed that the domains related to patient factors and those associated with incentives and resources had a significant effect on adherence to clinical practice guidelines (p = 0.000 and p = 0.015, respectively). There was also some influence of individual health professional factors (p = 0.081), as the responses of the administrative group differed from those of the health team (Table C in S3 Appendix).

Regarding the perception of GACIPD adherence determinants based on years of experience, the bivariate analysis did not show any significant association; see Table 5 (Table C in S3 Appendix).

**Table 5:** Likert scale statistical analysis of the perception of the GACIPD adherence determinants, according to professional profile. Online Questionnaire Urabá 2021.

DOMAIN*	Range**	Total	%	Physician n = 37	%	Nurse n = 21	%	Microbiologist n = 6	%	Nursing technicians n = 68	%	Administrative n = 4	%	Mean (SD)	KW***. (p-value)
**DA**	**18–42 LOW**	13	9.6	2	5.4	1	4.8	0	0	9	13.2	1	25.0	66.7 (14.0)	8.5 (0.129)
	**43–66 MEDIUM**	40	29.4	11	29.7	8	38.1	2	33.3	17	25.0	2	50.0		
	**67–90 HIGH**	83	61.0	24	64.9	12	57.1	4	66.7	42	61.8	1	25.0		
**DB**	**6–14 LOW**	32	23.5	11	29.7	5	23.8	1	16.7	13	19.1	2	50.0	18.31 (5.8)	4.2 (0.519)
	**15–21 MEDIUM**	53	39.0	12	32.4	10	47.6	4	66.7	25	36.8	2	50.0		
	**22–30 HIGH**	51	37.5	14	37.8	6	28.6	1	16.7	30	44.1	0	0.0		
**DC**	**5–11 LOW**	11	8.1	2	5.4	1	4.8	0	0.0	6	8.8	2	50.0	17.5 (4.0)	11.2 (0.047)
	**12–18 MEDIUM**	61	44.9	24	64.9	8	38.1	2	33.3	26	38.2	1	25.0		
	**19–25 HIGH**	64	47.1	11	29.7	12	57.1	4	66.7	36	52.9	1	25.0		
**DD**	**2–4 LOW**	17	12.5	3	8.1	3	14.3	0	0.0	9	13.2	2	50.0	6.2 (1.7)	3.8 (0.565)
	**5–6 MEDIUM**	67	49.3	22	59.5	11	52.4	4	66.7	29	42.6	1	25.0		
	**7–10 HIGH**	52	38.2	12	32.4	7	33.3	2	33.3	30	44.1	1	25.0		
**DE**	**7–16 LOW**	20	14.7	7	18.9	1	4.8	1	16.7	10	14.7	1	25.0	23.9 (6.1)	7.9 (0.159)
	**17–25 MEDIUM**	48	35.3	7	18.9	12	57.1	3	50.0	25	36.8	1	25.0		
	**26–35 HIGH**	68	50.0	23	62.2	8	38.1	2	33.3	33	48.5	2	50.0		
**DF**	**4–9 LOW**	26	19.1	9	24.3	3	14.3	0	0	13	19.1	1	25.0	12.9 (4.1)	8.4 (0.131)
	**10–15 MEDIUM**	63	46.3	20	54.1	11	52.4	3	50	27	39.7	2	50.0		
	**16–20 HIGH**	47	34.6	8	21.6	7	33.3	3	50	28	41.2	1	25.0		
**DG**	**2–4 LOW**	31	22.8	9	24.3	2	9.5	2	33.3	16	23.5	2	50.0	5.9 (2.1)	4.4 (0.492)
	**5–6 MEDIUM**	52	38.2	17	45.9	11	52.4	1	16.7	22	32.4	1	25.0		
	**7–10 HIGH**	53	39.0	11	29.7	8	38.1	3	50.0	30	44.1	1	25.0		
**TOTAL**	**44–103 LOW**	10	7.4	3	8.1	1	4.8	4	66.7	8	11.8	2	50.0	151.3 (31.4)	8.9 (0.111)
	**104–162 MEDIUM**	72	52.9	21	56.8	11	52.4	1	16.7	30	44.1	1	25.0		
	**163–220 HIGH**	54	39.7	13	35.1	9	42.9	1	16.7	30	44.1	1	25.0		

* DA: Determinants of guideline use; DB: Individual health professional factors; DC: Patient factors; DD: Professional interaction factors; DE: Incentive and resource factors; DF: Capacity for organizational change; DG: Social, political and legal factors.

**Score range: Minimum and maximum score for each domain stratified into categories

***Kruskal Wallis: Statistical association test of GACIPD adherence determinants, according to professional profile. Online Questionnaire Urabá 2021.

The convergence of the results of the quantitative and qualitative analysis is shown in Table A in S6 Appendix).

### Barriers and facilitators

Through a mixed-method study that combines qualitative and quantitative approaches, involving 246 officials including health workers, academic representatives, and decision-makers, the main barriers and facilitators for GACIPD adherence were identified. These are summarized in Table 6 (Tables A and B in S1 Tables and A-G in S2 Tables).

**Table 6:** Barriers and facilitators for the implementation of the GACIPD. Urabá 2021.

BARRIERS	FACILITATORS
Guide Indicative Factors	
• The guidelines are not clear regarding co-infections. • Difficulty in the diagnostic approach due to the different differential diagnoses of infectious febrile syndromes endemic in the region of Urabá. • Limitation of the guide regarding the management of co-infections, there is no adoption or adaptation of the guide in these circumstances.	• The guide is practical, simple, clear, user-friendly, complete, documented, and easy to implement. • Easy access to the guide through technological resources. • The guideline is clear regarding comorbidities. • Accumulated medical knowledge from practicing in a dengue-endemic area. • Easy access of patients from Apartadó to hospital facilities • Easy access to the Guidelines through the web
Individual factors of health professionals	
• Lack of interest of health personnel in self-learning and knowledge of the guidelines. • Lack of motivation at the personal level in terms of updating knowledge, and compliance with the recommendations of the guide. • Inexperience and unfamiliarity with the endemic diseases of the region, with which new professionals arrive. • Surveillance and lack of knowledge of the guidelines: not knowing how to fill out the notification form and not knowing the warning signs that warrant hospitalization.	• Motivation to follow up on implementation. • Conducting training in health posts far from the institution. • “I take care of myself at home" program
Patient factors	
• The guide does not include a context for the reality of our communities; there is a lack of emphasis on prevention measures from the community and families. • Non-compliance of nursing staff instructions by the relatives of patients with dengue fever. • Failure of the patient to recognize the warning signs or to follow up on the recommendations given by the health personnel to the patient and their family. • Lack of prevention strategies and community education. • Management of the patient’s beliefs regarding treatment and medical care, especially with the indigenous population.	• Communication between the nursing staff and the patient’s guardians about medical behavior, controls, promotion, and prevention of dengue. • The personal and institutional commitment to patient follow-up and health education for the community.
Professional interactions	
• We all work in isolation, each institution on its own, and there is a lack of an action route that is truly complied with at the municipal level so that there is efficiency in the health sector. • Difficulties and delays in the referral process. • The referral and the counter-referral process do not always work well, often patients who require a higher level of care end up being transferred between institutions without the approval of the receiving institution.	• Interdisciplinary work both in the processes of filling out the form and notification as well as in the processes of performing paraclinical tests and results. • Teamwork in the institution and good medical coordination, assertive communication regardless of the professional level.
Incentives and resources	
• Barriers on the administrative side to access the NS1 antigen test. • Lack of incentives for adherence to the guidelines. • Lack of rapid dengue testing in rural areas where, due to geographic and economic difficulties, patient diagnosis and treatment are delayed.	• Having the necessary resources for comprehensive and complete care: higher complexity institution, with the availability of different specialties and availability of adult ICU. • The personal satisfaction that health personnel have when providing care to a sick patient and seeing them recover.
Capacity for organizational change	
• Quality-management and follow-up auditing of guideline parameters is perceived as burdensome and persecutory rather than friendly and sensitive. • Institutional policies based on production and not on the quality of health personnel. • Lack of a solid institutional guideline that is not affected by frequent changes in the institution’s administration. • Frequent management changes that interfere with the continuity of processes. • Continuous change of medical staff • Lack of institutional policies and follow-up to the guideline adherence process. • Low number of health personnel for the high flow of patients in the institution and high workload.	• Being periodically trained by the institution. • The availability of the guide on the institutional platform • There is orientation and socialization for the patient on warning signs, the importance of fallow up appointments, form of dengue transmission, and prevention. • Standardization of processes and unification of knowledge for its application in patient care. • Institutional support from promotion and prevention, surveillance, nursing, and quality, with early alerts and follow-up of clinical history and cases.
Social, political, and legal factors	
• Limitations in timely access to health care services due to geographic and economic barriers. • Unavailability of pediatric ICU in the area. • Limitations for the care of the uninsured population (migrants, indigenous, indigent, etc.). • Legislation and regulations that slow down the process of updating guidelines.	

Regarding the use of the guideline, 86.7% of participants reported having heard of the clinical practice guideline, and 88.9% believed that the guideline optimizes health care delivery and outcomes by supporting patient-clinician communication and decision-making.

In terms of organizational capacity for change, the results indicate that only 3.6% of the surveyed individuals believed that their institution adjusts the prioritization of adequate care in accordance with the guideline; more than 50% of the health institutions lack clear institutional guidelines for the socialization, implementation, and follow-up of the GACIPD (Table A in S6 Appendix).

Regarding resources and incentives, 63.2% of professionals expressed that financial, human, structural resources or supplies are necessary to ensure GACIPD adherence for comprehensive clinical care of patients with dengue. However, other officials mentioned the scarcity of personnel to support institutional management in terms of adherence to the dengue guidelines, such as the lack of rapid tests for dengue and limited access to blood counts, particularly at the primary levels of care (Table A in S6 Appendix).

In terms of professional interactions, approximately 44.8% of the participants agree on the existence of effective referral and counter-referral between levels of care. This suggests that collaboration and communication among healthcare professionals from different disciplines are happening to some extent (Table A in S6 Appendix).

In the domain of individual factors, the results highlight the importance of individual attitudes and experiences. Only 10% of professionals completely agree that their work is in accordance with the GACIPD. However, 48.5% agree that they adopt the guide, indicating some level of adherence. Additionally, 44.8% agree that the continuous education they have received in the guidelines has been effective (Table B in S4 Appendix).

Regarding patient factors, 50% of healthcare professionals agree that patient beliefs or knowledge influence medical care, and 64.7% agree that medical care enables patients to adhere to the recommendations outlined in the guideline (Table A in S6 Appendix).

In the domain of social, political, and legal factors, 60.2% of the participants agree that institutional policies facilitate adherence to the guideline. However, only 8.8% completely agree that the economic conditions of their institution affect guideline adherence. Half of the respondents report agreeing that the contract under which they work for their institution facilitates guideline adherence (Table A in S6 Appendix).

## Discussion

The study highlights several important points. Firstly, it reveals a lack of consensus among healthcare professionals and decision-makers regarding the determinants of GACIPD adherence. This indicates the need for additional efforts to align perspectives and promote a shared understanding of its importance.

This study had some limitations due to the Covid-19 emergency, much of it had to be carried out virtually. Two virtual focus groups may have had limited participation due to poor internet connectivity. Due to administrative restrictions, focus groups were intentionally held in a single time slot at each institution, reducing participation, and excluding input from personnel working in other shifts who were not in attendance at that time. Work overload due to the pandemic might have negatively impacted the willingness of healthcare professionals to take the time to participate in the study. We are going through a transition period between the update of the Pan American Health Organization’s dengue guidelines in 2015 and the Colombian guidelines from 2010 that have not been updated. Our national guideline lacks a differential approach aimed at each of the different health workers (physicians, nurses, microbiologists, medical specialists) and does not discriminate in accordance with health care level or community prevention.

A key finding was the perception of the guidelines themselves. While professionals generally agree that the GACIPD is an easy and accessible tool, there are gaps in its practical application. This suggests the need for better integration and dissemination of the guideline within healthcare systems. It is recommended to prioritize the inclusion of evidence-based medicine (EBM) in the academic curriculum. By incorporating training on the GACIPD during clinical education, future healthcare professionals can develop the skills and confidence necessary to effectively apply the guideline in practice.

The importance of technology in facilitating access to GACIPD is emphasized. Ensuring the provision of user-friendly and accessible guides or applications would enable healthcare professionals to access guidelines in real time, supporting their decision-making process and improving adherence to recommended practices. The need for joint efforts by academic institutions, healthcare organizations, and policymakers is emphasized to improve the understanding, acceptance, and implementation of the GACIPD.

The determinant of patient factors was significant in the negative perception of adherence to GACIPD. There is a consensus that the GACIPD does not adequately emphasize patient education. Participants suggest the importance of incorporating a community educational component into the guideline to better address the needs and understanding of patients and their families. This highlights the need to adopt a more comprehensive approach, centered around community engagement, for its development and implementation.

While the importance of effective referral and counter-referral systems is recognized, the complexity of the healthcare system and contractual agreements with healthcare service providers (EPS) pose challenges. At times, the sustainability of the system takes priority over patient care, and there are difficulties in referring patients to distant municipalities. This indicates the need to improve coordination and communication among different levels of care.

The development and implementation of a clinical practice guideline like the GACIPD are considered costly, and there is a gap in investment compared to other priority areas. Addressing these challenges would require a comprehensive approach that aligns resources and incentives with the goals of guideline adherence and improving patient care.

However, satisfactory results are expected, as was achieved in India, with resident physicians who received adequate training in the guideline during the low dengue season and were able to impact the mortality indicator by 7.1%, with 0 deaths after the intervention [23]. Similarly in a Malaysian teaching hospital, after grouping all patients in an infectious diseases service, the authors conclude that the cohort of adult dengue patients under a dedicated and trained team of physicians and nurses led to substantial improvement in the quality of care and clinical outcome [24].

The focus groups identified barriers related to institutional and individual factors, such as institutional policies, quality management systems, socialization, implementation, and monitoring GACIPD adherence. Although the guideline remains accessible to everyone in the emergency department, most professionals are limited by their knowledge of diagnosing and managing patients with dengue.

Challenges arise when coordinating care across different levels of healthcare. Referral and counter-referral processes require better harmonization and communication to ensure efficient and timely transfers of patients. The lack of a clear action plan can contribute to inefficiencies in the healthcare system. The lack of clear policies, monitoring, and incentives to strengthen adherence to guidelines is evident, which can hinder their effective implementation. The focus on production rather than quality, along with structural and financial difficulties in the healthcare system, further exacerbates the challenges.

In general, the participants indicate that they have comprehensive and easy-to-implement guidelines for dengue care. However, they highlight some limitations regarding coinfections. Users in Colombia, for example, had limited knowledge of the guidelines for arbovirus diagnosis and management [25], while a study in Ecuador, reported that WHO guidelines were helpful in classifying and managing patients with dengue [26]. In Malaysia, better adherence to dengue guidelines was observed in hospitals compared to primary care levels [27].

Significant variations were found in adherence to different aspects of the guidelines, with the completion of the notification form being the aspect with the highest adherence and the identification of hemodynamic status being the aspect with the lowest adherence. While the use of the guidelines is generally positive, there is room for improvement in training among healthcare professionals, and adaptations may be necessary to account for endemic conditions.

## Conclusions

The capacity for organizational change is highlighted as the major barrier encountered, specifically the absence of institutional policies and incentives to strengthen the adherence process to the guidelines in some healthcare institutions. There is a lack of administrative commitment to maintaining quality standards in the socialization, implementation, and monitoring processes of dengue guideline adherence.

Most participants affirm that they have a comprehensive, clear, useful, easily accessible, and well-documented guideline. Despite this, they admitted having limited knowledge of it. Educational strategies could be implemented to reinforce the impact of the guideline as a facilitator and reduce the lack of knowledge around it as a barrier. They highlight that the guideline is comprehensive for comorbidities but does not focus on febrile syndromes responsible for coinfections with other tropical diseases.

Decision-makers and academics highlighted the need to enhance the teaching and dissemination of national and regional clinical guidelines in undergraduate and postgraduate programs. Healthcare professionals in focus groups and surveys acknowledged the benefits of the clinical guideline and emphasized the need to strengthen interdisciplinary work, standardize the pathways at different levels of care, and all administrative processes. Administrative personnel emphasized that there is a lack of educational tools to guide the community in primary and secondary prevention actions.

Overall, this study provides valuable insights into the determinants of adherence to the guidelines for dengue care. It highlights the need for institutional support, clear policies, and resources, as well as improvements in patient-centered approaches, professional interactions, and adaptation to local contexts. Addressing these factors can contribute to better implementation and adherence to the guidelines, ultimately improving the quality of care for patients with dengue.

### Contributions to the literature

• Health institutions must take responsibility for strengthening institutional guidelines to ensure adherence and knowledge appropriation by healthcare personnel regarding the Comprehensive Care Guide for Patients with Dengue (GACIPD).

• Despite being considered comprehensive, well-documented, and easy to implement, the GACIPD should be enhanced to include the management of acute febrile syndromes and coinfections with prevalent tropical diseases in the Urabá region.

## Supporting information

S1 Tables Table A. IPS Barriers Facilitators. Table B. Consolidate Health Personal Barriers and Facilitators.
(XLSX)

S2 Tables Table A. Decision-maker 1 Barriers and Facilitators. Table B. Academic 1 Barriers and Facilitators. Table C. Academic 2 Barriers and Facilitators. Table D. Academic 3 Barriers and Facilitators. Table E. Academic 4 Barriers and Facilitators. Table F. Consolidated everything Barriers and Facilitators. Table G. Consolidated summary Barriers and Facilitators.
(XLSX)

S1 Appendix Fig A. Diagram of the study design. Table A. Overview of ICTD domains and determinants. Semi-structured Interview. Online questionnaire. Validation of semi-structured interview questions/structured questionnaire for health personal.
(DOCX)

S2 Appendix Table A. Consolidated criteria for reporting qualitative studies (COREQ): 32-item checklist. Table B. STROBE Statement—checklist of items that should be included in reports of observational studies.
(DOC)

S3 Appendix Table A. Statistical analysis of the perception of the determinants of adherence to the GACIPD according to each domain. Table B. Statistical analysis of the perception of the determinants of adherence to the GACIPD, according to time of professional experience. Urabá 2021 online survey. Table C. Statistical analysis of the perception of the determinants of adherence to the GACIPD, according to time of professional experience. Urabá 2021 online survey.
(DOCX)

S4 Appendix Table A. Results of the semi-structured interviews and focus groups. Table B. Results of focus groups. Table C. Results of the semi-structured interviews.
(DOCX)

S5 Appendix Distribution of the components of each of the 7 domains and score according to Likert scale. Domain A. Guide determinants. Domain B. Individual factors of health professionals. Domain C. Patient factors. Domain D. Professional Interactions. Domain E. Incentives and resources. Domain F. Capacity for organizational change. Domain G. Social, political, and legal factors.
(DOCX)

S6 Appendix Table A. Visualization of quantitative and qualitative results for determinants of GACIPD adherence. Urabá 2021.
(DOCX)
