# Supplementary material for: Practice determinants for adherence to the Guide for the Comprehensive Clinical Care of Dengue Patients, Urabá (Colombia). A multifaceted approach to implementation research
Source: PLoS Negl Trop Dis. 2024 Aug 15;18(8):e0012361. doi: 10.1371/journal.pntd.0012361 (PMC11349210; doi:10.1371/journal.pntd.0012361)
Supplement: S1 Appendix — Fig A. Diagram of the study design. Table A. Overview of ICTD domains and determinants. Semi-structured Interview. Online questionnaire. Validation of semi-structured interview questions/structured questionnaire for health personal. (DOCX) [file pntd.0012361.s003.docx]

**Annex 1: Supplementary Material S1**

**1. Fig A. Diagram of the study design adapted from Apodaca et al., (1).**

**Qualitative**

**Semi-structured Interviews**

**Focus Groups**

**Quantitative**

**On-line questionnaire**

**TICD - GACIPD**

**Data collection**

**Data collection**

**Decision makers**

**Ministry of Health**

**Academics**

**Health professionals**

**Health professionals from health institutions in 4 municipalities**

**Narrative extraction and analysis**

**Analysis and factors of association**

**Data analysis**

**Data analysis**

**Results**

**Results**

**Determinant**

**Barriers and facilitators**

Triangulation

Compare, contrast and interpretation

Quali + quanti


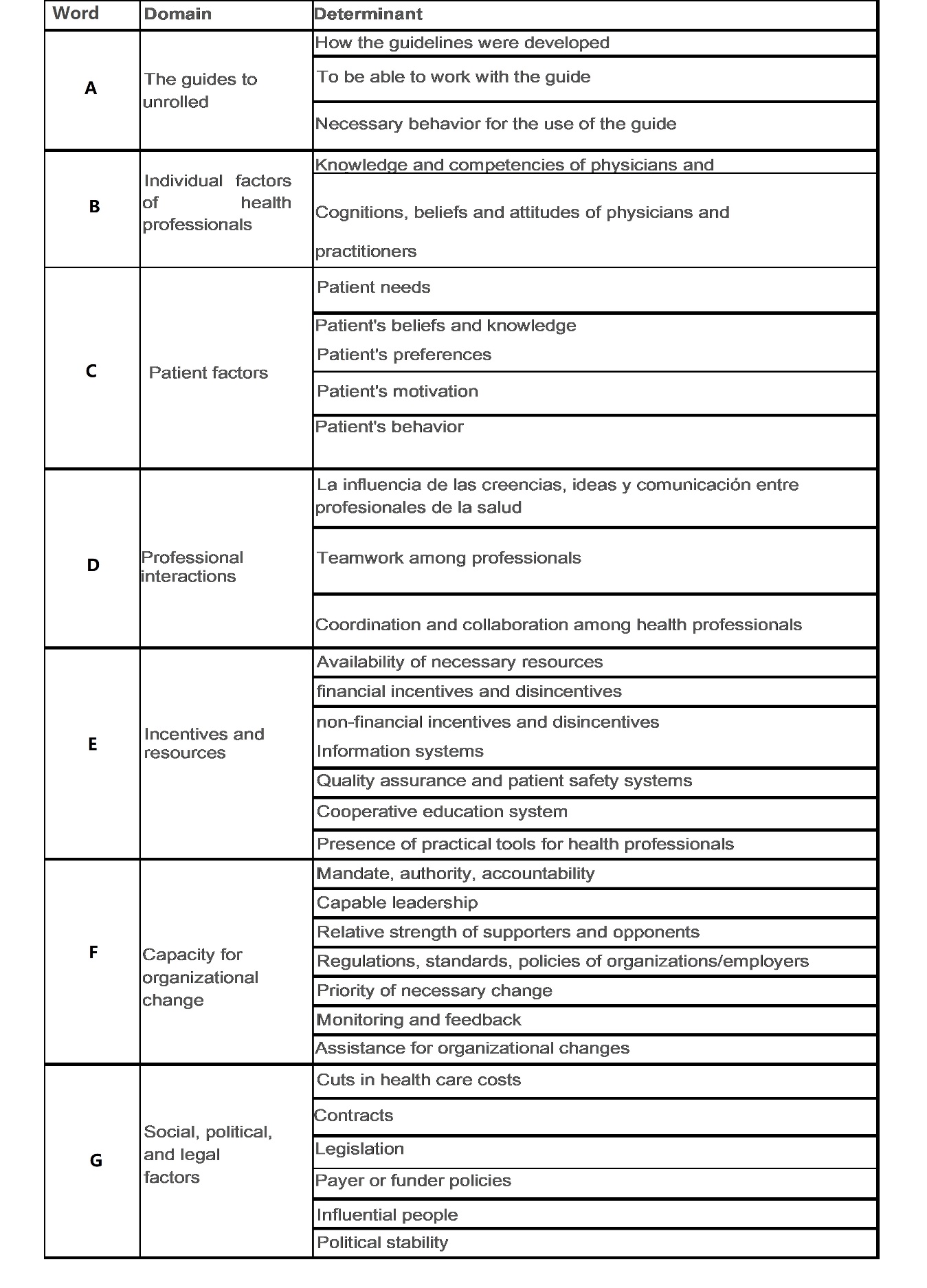
**2. Table A. Overview of ICTD domains and determinants adapted from Flottorp et al., (2)**

**3. Semi-structured Interview**

**"Strengthening of capacities for prevention, detection, diagnosis, treatment and follow-up of dengue cases in municipalities of the banana axis (Turbo, Apartadó, Carepa and Chigorodó) with high morbimortality rates. Urabá 2020-2022".**

**Adherence to Dengue Guidelines Project**

**Semi-structured interview / focus group to assess adherence to guidelines by health personnel.**

I. Objective: to identify barriers and facilitators in the adherence to comprehensive dengue management guidelines, based on the different clinical practice domains, by academic representatives, decision-makers and health personnel.

II. Preparation: After a collaboration between co-researchers and an expert consultant in qualitative research, a guide was defined for conducting the semi-structured interview with representatives of the academic sector.

Number of participants: 1 person per interview

Date: date and time of the meeting.

Duration of the session: 60 minutes

Recording of information: Note taking and audio recording.

Ethical considerations: acquisition of informed consent for the use of information.

Type of research process: no risk

Place: Academic institutions via virtual meetings.

**Process**

Presentation of the day and the participants

Signing of informed consent form

Conducting the interview

Closing and farewell

Interview guide

1. What is your opinion of the Colombian guide for the comprehensive clinical care of patients with dengue fever?

2. What is your experience with the use of the GACIPD?

3. Have you received training on the use of the GACIPD?

4. What is your opinion of the recommendations in the guide (appropriate for the region of Urabá).

5. How do you rate the implementation of the GACIPD in the country for the care of patients?

6. Do you consider the GACIPD a support to the professionals in their clinical practice, or on the contrary, is it a barrier?

7. What is the process of GACIPD appropriation by health personnel for the clinical care of patients?

8. What are the advantages and benefits of working in coordination with the GACIPD?

9. Is there communication between the distinct levels of care (referral, counter-referral and primary transfer) and health professionals?

10. Do you know and apply the audit guide in the care of patients with dengue fever?

11. What are the financial, human, and operational resources that facilitate adherence to the GACIPD?

12. How do you coordinate with the Ministry of Health for the implementation of the GACIPD?

13. How do social, political, and legal factors (internal and external) influence the implementation of the GACIPD?

14. Do you believe that the GACIPD responds to the needs of dengue prevention in communities?

**4.** **Online questionnaire**

# “Strengthening of capacities for prevention, detection, diagnosis, treatment, and follow-up of dengue cases, in municipalities of the banana axis (Turbo, Apartadó, Carepa and Chigorodó) that exhibit high morbimortality rates. Urabá 2020-2022".

**Adherence to dengue guidelines Project**

**Structured questionnaire to evaluate**

**adherence to guidelines, health personnel.**

**Objective:** to identify barriers or facilitators in the adherence to the "Guide for the comprehensive clinical care of patients with dengue" in the region of Urabá Antioqueño based on domains.

**Procedure:** In the following questionnaire you will find some questions for you, as health personnel in the exercise of your profession, to identify barriers and facilitators.

**Clinical Orientation**

**Determinants Questionnaire**

# SECTION 1. BACKGROUND INFORMATION

| Sex: | - Female Male |
| --- | --- |
| Profession/Speciality/Subspeciality: |  |
| Years of professional experience: | -------- years |
| Institution to which the participant belongs:  ¿ Have you heard about clinical practice guidelines?  ¿ Do you believe that clinical practice guidelines (in general) optimize care delivery and outcomes by supporting patient-clinical communication and decision making? | - Yes No  Not sure. - Yes No  Not sure |
| ¿ Have you participated in the development of a clinical practice guideline or other guidelines.? | - Yes No Not sure |

**SECTION 2. DETERMINANTS OF GUIDELINES USE**

1. ¿ What is your familiarity with the "Colombian Guide for the comprehensive clinical care of patients with dengue fever"?

*Choose the answer that best fits your scenario.*

- I was not aware of this guideline prior to this questionnaire.
- I am aware of the GACIPD, but I have not read it.
- I have read it once and never again.
- I have read it on multiple occasions.
- I have read it and put it into practice.

1. ¿ What is your actual use of the "Guideline for the comprehensive clinical care of patients with dengue fever"?

*Choose the answer that best fits your scenario.*

- I have never used the GACIPD and do not plan to use it.
- I have never used the GACIPD, but I will consider using it.
- I have never used the GACIPD, but I will use it.
- I have used the GACIPD only once.
- I have used the GACIPD a couple of times.
- I have used the GACIPD extensively.

1. In your clinical practice has someone demanded that you use the GACIPD and the actions or activities recommended therein:

*Choose all that apply.*

- Patients
- Colleagues
- Managers or executives of my organization
- Medical audit and quality management system
- Territorial entity
- Professional Society
- No one

*Choose the answer that best fits your scenario.*

1. I agree with the contents of the GACIPD

| Strongly disagree | Disagree | Neither agree nor disagree | Agree | Strongly  Agree | Not sure |
| --- | --- | --- | --- | --- | --- |
|  |  |  |  |  |  |

1. Following the GACIPD will improve the delivery of care

| Strongly disagree | Disagree | Neither agree nor disagree | Agree | Strongly  Agree | Not sure |
| --- | --- | --- | --- | --- | --- |
|  |  |  |  |  |  |

1. Following the GACIPD will improve patients' clinical outcome

| Strongly disagree | Disagree | Neither agree nor disagree | Agree | Strongly  Agree | Not sure |
| --- | --- | --- | --- | --- | --- |
|  |  |  |  |  |  |

1. Following the GACIPD has advantages for my professional practice at the institution

| Strongly disagree | Disagree | Neither agree nor disagree | Agree | Strongly  Agree | Not sure |
| --- | --- | --- | --- | --- | --- |
|  |  |  |  |  |  |

1. I have the general knowledge of dengue needed to use the GACIPD.

| Strongly disagree | Disagree | Neither agree nor disagree | Agree | Strongly  Agree | Not sure |
| --- | --- | --- | --- | --- | --- |
|  |  |  |  |  |  |

1. I am trained in the skills (technical, procedural, cognitive, etc.) needed to use the GACIPD.

| Strongly disagree | Disagree | Neither agree nor disagree | Agree | Strongly  Agree | Not sure |
| --- | --- | --- | --- | --- | --- |
|  |  |  |  |  |  |

1. I identify following the procedures, actions or activities recommended in the GACIPD among my professional responsibilities.

| Strongly disagree | Disagree | Neither agree nor disagree | Agree | Strongly  Agree | Not sure |
| --- | --- | --- | --- | --- | --- |
|  |  |  |  |  |  |

1. I have the autonomy to make the necessary changes in institutional policies to follow the GACIPD.

| Strongly disagree | Disagree | Neither agree nor disagree | Agree | Strongly  Agree | Not sure |
| --- | --- | --- | --- | --- | --- |
|  |  |  |  |  |  |

1. My institution provides support (leadership, resources, assistance, time, etc.) needed to use the GACIPD.

| Strongly disagree | Disagree | Neither agree nor disagree | Agree | Strongly  Agree | Not sure |
| --- | --- | --- | --- | --- | --- |
|  |  |  |  |  |  |

1. The procedures, actions or activities recommended in the GACIPD are easy to incorporate into my practice.

| Strongly disagree | Disagree | Neither agree nor disagree | Agree | Strongly  Agree | Not sure |
| --- | --- | --- | --- | --- | --- |
|  |  |  |  |  |  |

1. It is easy to find information about dengue fever in the GACIPD.

| Strongly disagree | Disagree | Neither agree nor disagree | Agree | Strongly  Agree | Not sure |
| --- | --- | --- | --- | --- | --- |
|  |  |  |  |  |  |

1. Information on GACIPD recommendations for dengue care is clear and unambiguous.

| Strongly disagree | Disagree | Neither agree nor disagree | Agree | Strongly  Agree | Not sure |
| --- | --- | --- | --- | --- | --- |
|  |  |  |  |  |  |

1. The GACIPD includes implementation tools (clinical summary, patient summary, algorithm, medical record forms, etc.) that are useful for professional practice.

| Strongly disagree | Disagree | Neither agree nor disagree | Agree | Strongly  Agree | Not sure |
| --- | --- | --- | --- | --- | --- |
|  |  |  |  |  |  |

1. The GACIPD clearly describes the laboratory tests that support the recommendations.

| Strongly disagree | Disagree | Neither agree nor disagree | Agree | Strongly  Agree | Not sure |
| --- | --- | --- | --- | --- | --- |
|  |  |  |  |  |  |

1. The GACIPD is consistent with the available evidence.

| Strongly disagree | Disagree | Neither agree nor disagree | Agree | Strongly  Agree | Not sure |
| --- | --- | --- | --- | --- | --- |
|  |  |  |  |  |  |

**SECTION 3. MANAGERIAL / ORIENTATIONAL FACTORS**

1. The Guide for the comprehensive clinical care of patients with dengue fever provides sufficient detail so that the activities set out in it can be adapted.

| Strongly disagree | Disagree | Neither agree nor disagree | Agree | Strongly  Agree | Not sure |
| --- | --- | --- | --- | --- | --- |
|  |  |  |  |  |  |

1. Accessing the GACIPD is easy.

| Strongly disagree | Disagree | Neither agree nor disagree | Agree | Strongly  Agree | Not sure |
| --- | --- | --- | --- | --- | --- |
|  |  |  |  |  |  |

1. The GACIPD is feasible and accessible for timely medical care at all levels of care.

| Strongly disagree | Disagree | Neither agree nor disagree | Agree | Strongly  Agree | Not sure |
| --- | --- | --- | --- | --- | --- |
|  |  |  |  |  |  |

1. ¿ Do you consider that the care you provide as a health professional is in accordance with the GACIPD?

| Strongly disagree | Disagree | Neither agree nor disagree | Agree | Strongly  Agree | Not sure |
| --- | --- | --- | --- | --- | --- |
|  |  |  |  |  |  |

1. Adherence to the GACIPD has some degree of difficulty.

| Strongly disagree | Disagree | Neither agree nor disagree | Agree | Strongly  Agree | Not sure |
| --- | --- | --- | --- | --- | --- |
|  |  |  |  |  |  |

**SECTION 4. INDIVIDUAL FACTORS OF HEALTH PROFESSIONALS**

The following questions explore the understanding you have of your colleagues' professional practice.

1. Health professionals are familiar with the GACIPD

| Strongly disagree | Disagree | Neither agree nor disagree | Agree | Strongly  Agree | Not sure |
| --- | --- | --- | --- | --- | --- |
|  |  |  |  |  |  |

1. Knowledge or experience of the GACIPD affects adherence to the RIAS (Ruta de Atención Integral en Salud) for Dengue.

| Strongly disagree | Disagree | Neither agree nor disagree | Agree | Strongly  Agree | Not sure |
| --- | --- | --- | --- | --- | --- |
|  |  |  |  |  |  |

1. Health professionals agree with the GACIPD

| Strongly disagree | Disagree | Neither agree nor disagree | Agree | Strongly  Agree | Not sure |
| --- | --- | --- | --- | --- | --- |
|  |  |  |  |  |  |

1. Health professionals adopt the GACIPD

| Strongly disagree | Disagree | Neither agree nor disagree | Agree | Strongly  Agree | Not sure |
| --- | --- | --- | --- | --- | --- |
|  |  |  |  |  |  |

1. Continuing education in the GACIPD has been effective.

| Strongly disagree | Disagree | Neither agree nor disagree | Agree | Strongly  Agree | Not sure |
| --- | --- | --- | --- | --- | --- |
|  |  |  |  |  |  |

**SECTION 5. PATIENT FACTORS**

1. What is your level of knowledge of the GACIPD recommendations for patients?

| Strongly disagree | Disagree | Neither agree nor disagree | Agree | Strongly  Agree | Not sure |
| --- | --- | --- | --- | --- | --- |
|  |  |  |  |  |  |

1. Patients' beliefs or knowledge influence medical care according to the GACIPD.

| Strongly disagree | Disagree | Neither agree nor disagree | Agree | Strongly  Agree | Not sure |
| --- | --- | --- | --- | --- | --- |
|  |  |  |  |  |  |

1. Medical care enables patients to adhere to the recommendations set forth in the GACIPD.

| Strongly disagree | Disagree | Neither agree nor disagree | Agree | Strongly  Agree | Not sure |
| --- | --- | --- | --- | --- | --- |
|  |  |  |  |  |  |

**SECTION 6. PROFESSIONAL INTERACTIONS**

1. Adoption of the GACIPD is influenced by organizational norms.

| Strongly disagree | Disagree | Neither agree nor disagree | Agree | Strongly  Agree | Not sure |
| --- | --- | --- | --- | --- | --- |
|  |  |  |  |  |  |

1. The GACIPD requires the interaction of health care teams.

| Strongly disagree | Disagree | Neither agree nor disagree | Agree | Strongly  Agree | Not sure |
| --- | --- | --- | --- | --- | --- |
|  |  |  |  |  |  |

1. There is effective referral and counter-referral between levels of care, in accordance with the recommendations of the GACIPD.

| Strongly disagree | Disagree | Neither agree nor disagree | Agree | Strongly  Agree | Not sure |
| --- | --- | --- | --- | --- | --- |
|  |  |  |  |  |  |

**SECTION 7. INCENTIVES AND RESOURCES**

1. Availability of resources (financial, human, structural or supplies) is required to ensure adherence to the GACIPD.

| Strongly disagree | Disagree | Neither agree nor disagree | Agree | Strongly  Agree | Not sure |
| --- | --- | --- | --- | --- | --- |
|  |  |  |  |  |  |

1. Incentives exist for healthcare professionals to ensure adherence to the GACIPD.

| Strongly disagree | Disagree | Neither agree nor disagree | Agree | Strongly  Agree | Not sure |
| --- | --- | --- | --- | --- | --- |
|  |  |  |  |  |  |

1. The institutional information system facilitates adherence to the GACIPD.

| Strongly disagree | Disagree | Neither agree nor disagree | Agree | Strongly  Agree | Not sure |
| --- | --- | --- | --- | --- | --- |
|  |  |  |  |  |  |

1. The institution has a quality management system aimed at patient safety in accordance with the GACIPD.

| Strongly disagree | Disagree | Neither agree nor disagree | Agree | Strongly  Agree | Not sure |
| --- | --- | --- | --- | --- | --- |
|  |  |  |  |  |  |

1. There is continuing education in the institution to adapt the implementation strategy of the GACIPD.

| Strongly disagree | Disagree | Neither agree nor disagree | Agree | Strongly  Agree | Not sure |
| --- | --- | --- | --- | --- | --- |
|  |  |  |  |  |  |

1. Physicians have decision-making authority in relation to the GACIPD.

| Strongly disagree | Disagree | Neither agree nor disagree | Agree | Strongly  Agree | Not sure |
| --- | --- | --- | --- | --- | --- |
|  |  |  |  |  |  |

# SECTION 8. CAPACITY FOR ORGANIZATIONAL CHANGE

1. Organizational changes are required to ensure adherence to the GACIPD.

| Strongly disagree | Disagree | Neither agree nor disagree | Agree | Strongly  Agree | Not sure |
| --- | --- | --- | --- | --- | --- |
|  |  |  |  |  |  |

1. Institutional managers have the capacity to amend and improve adherence to the GACIPD.

| Strongly disagree | Disagree | Neither agree nor disagree | Agree | Strongly  Agree | Not sure |
| --- | --- | --- | --- | --- | --- |
|  |  |  |  |  |  |

1. Institutional guidelines or policies facilitate the necessary changes for the adoption of the GACIPD.

| Strongly disagree | Disagree | Neither agree nor disagree | Agree | Strongly  Agree | Not sure |
| --- | --- | --- | --- | --- | --- |
|  |  |  |  |  |  |

1. In your institution, the prioritization of needs is adjusted in accordance with the GACIPD.

| Strongly disagree | Disagree | Neither agree nor disagree | Agree | Strongly  Agree | Not sure |
| --- | --- | --- | --- | --- | --- |
|  |  |  |  |  |  |

1. Implementation of the GACIPD is being monitored at your institution.

| Strongly disagree | Disagree | Neither agree nor disagree | Agree | Strongly  Agree | Not sure |
| --- | --- | --- | --- | --- | --- |
|  |  |  |  |  |  |

1. In your institution, external advice is required to make the necessary changes in order to adhere to the GACIPD.

| Strongly disagree | Disagree | Neither agree nor disagree | Agree | Strongly  Agree | Not sure |
| --- | --- | --- | --- | --- | --- |
|  |  |  |  |  |  |

**SECTION 9. SOCIAL, POLITICAL AND LEGAL FACTORS**

1. Institutional policies facilitate adherence to the GACIPD.

| Strongly disagree | Disagree | Neither agree nor disagree | Agree | Strongly  Agree | Not sure |
| --- | --- | --- | --- | --- | --- |
|  |  |  |  |  |  |

1. The economic conditions of your institution affect adherence to the GACIPD.

| Strongly disagree | Disagree | Neither agree nor disagree | Agree | Strongly  Agree | Not sure |
| --- | --- | --- | --- | --- | --- |
|  |  |  |  |  |  |

1. Your type of contract with the institution facilitates adherence to the GACIPD.

| Strongly disagree | Disagree | Neither agree nor disagree | Agree | Strongly  Agree | Not sure |
| --- | --- | --- | --- | --- | --- |
|  |  |  |  |  |  |

**SECTION 10. LEARNING STYLE**

1. ¿ What sources do you consult most often for knowledge to guide clinical decision making?

*Choose all that apply.*

- Colleagues
- Medical Literature
- Electronic application or database
- Internet
- Guidance from government, regulatory agency, or medical society
- Educational meetings/conferences
- Medical books
- Systematic reviews
- Other (specify):

1. ¿ How do you prefer to learn about clinical care guidelines.?

*Choose all that apply.*

- Educational meetings/conferences
- Online guide developer's website
- Guideline developer email
- Medical journal publication
- Webinars from other institutions
- Virtual courses
- Other (specify):

1. ¿ What is your preferred format for updating and continuing education processes?

*Choose all that apply.*

- Electronic version (software) on the computer
- Electronic version on the developer's website
- Hard copy
- Other (specify):

**5. VALIDATION OF SEMI-STRUCTURED INTERVIEW QUESTIONS/STRUCTURED QUESTIONNAIRE FOR HEALTH PERSONNEL**

**“Strengthening of capacities for prevention, detection, diagnosis, treatment and follow-up of dengue cases in municipalities of the banana axis (Turbo, Apartadó, Carepa and Chigorodó) with high morbimortality rates. Urabá 2020-2022”**

**Colombian Institute of Tropical Medicine ICMT - CES University - FESU – Minciencias**

**VALIDATION OF SEMI-STRUCTURED INTERVIEW QUESTIONS / STRUCTURED QUESTIONNAIRE FOR HEALTH PERSONNEL**

**Objective:** To validate and verify the consistency of the verification questions aimed at management and administrative personnel in the areas of healthcare and human talent training for the component of adherence to the guidelines for dengue management in the region of Urabá Antioqueño based on seven domains that evaluate barriers and facilitators.

**Procedure:** In the following instrument, you will find some questions so that you, as a facilitator of the process of this research protocol, can identify the consistency of the verification questions and their applicability.

Name(s): ____________________________________________________________

Date: _______________________

For each domain I evaluated the questions that seek to identify barriers and facilitators for the implementation of the dengue comprehensive care guide. The answers should reflect as closely as possible the domains to which they are responding (2,3).

In addition, complete each domain with comments (including explanations of perceived problems and suggestions for improvement).

1. **GUIDELINE FACTORS**
2. **INDIVIDUAL HEALTH PROFESSIONAL FACTORS**
3. **PATIENT FACTORS**
4. **PROFESSIONAL INTERACTIONS**
5. **INCENTIVES AND RESOURCES**
6. **CAPACITY FOR ORGANIZATIONAL CHANGE**
7. **SOCIAL, POLITICAL AND LEGAL FACTORS**

**Completeness**

Are potentially crucial factors missing from the verification questions?

| YES | NO | UNCERTAIN |
| --- | --- | --- |
|  |  |  |

Comments (including explanations of perceived problems and suggestions for improvements): __________________________________________________________________________________________

**Relevance**

Are there factors that are included in the verification questions that shouldn’t be?

| YES | NO | UNCERTAIN |
| --- | --- | --- |
|  |  |  |

Comments (including explanations of perceived problems and suggestions for improvements): _______________________________________________________________________________________

**Applicability**

Are the verification questions applicable across different settings (e.g., primary and secondary care) and different types of practices (including prevention, diagnosis and treatment for dengue)?

| YES | NO | UNCERTAIN |
| --- | --- | --- |
|  |  |  |

Comments (including explanations of perceived problems and suggestions for improvements): __________________________________________________________________________________________

**Simplicity**

Are the verification questions more complicated than necessary?

| YES | NO | UNCERTAIN |
| --- | --- | --- |
|  |  |  |

Comments (including explanations of perceived problems and suggestions for improvements): __________________________________________________________________________________________

**Logic**

Are the verification questions organized in a logical manner that is easy to understand?

| YES | NO | UNCERTAIN |
| --- | --- | --- |
|  |  |  |

Comments (including explanations of perceived problems and suggestions for improvements): __________________________________________________________________________________________

**Clarity**

Are factors and domains (groups of factors) labeled and explained in a way that is easy to understand?

| YES | NO | UNCERTAIN |
| --- | --- | --- |
|  |  |  |

Comments (including explanations of perceived problems and suggestions for improvements):

__________________________________________________________________________________________

**Usability**

Would it be easy for implementation researchers to use the verification questions?

| YES | NO | UNCERTAIN |
| --- | --- | --- |
|  |  |  |

Comments (including explanations of perceived problems and suggestions for improvements): __________________________________________________________________________________________

Would it be easy for non-implementation researchers to use the verification questions?

| YES | NO | UNCERTAIN |
| --- | --- | --- |
|  |  |  |

Comments (including explanations of perceived problems and suggestions for improvements):

__________________________________________________________________________________________

**Adequacy**

Are the verification questions adequate to help people identify and prioritize determinants of change in practice that should be taken into account when designing implementation strategies?

| YES | NO | UNCERTAIN |
| --- | --- | --- |
|  |  |  |

Comments (including explanations of perceived problems and suggestions for improvements): __________________________________________________________________________________________

**Usefulness**

Are the verification questions likely to be useful to the people designing the implementation strategies?

| YES | NO | UNCERTAIN |
| --- | --- | --- |
|  |  |  |

Comments (including explanations of perceived problems and suggestions for improvements): __________________________________________________________________________________________

Are the verification questions likely to be useful for determinants of practice in research reports?

| YES | NO | UNCERTAIN |
| --- | --- | --- |
|  |  |  |

Comments (including explanations of perceived problems and suggestions for improvements): __________________________________________________________________________________________

**Overall evaluation**

After the evaluation of each domain, perform an overall evaluation of the questionnaire with the following parameters:

1. In general, are the verification questions useful in identifying and prioritizing?

Yes ____

Partially ____

No______

***Yes -*** *Could be used as-is with little or no modification.*

***Partially -*** *Needs some modification or further development.*

***No -*** *No suitable.*

**Strengths**

1. What do you like about the verification questions?

**Weaknesses**

**3.** What do you dislike about the verification questions and what suggestions do you have to improve them**?** _______________________________________________________________________________________

4. Include any other comments you have regarding the verification questions.

__________________________________________________________________________________________

**6. REFERENCES**

1. Apodaca Michel B, Navarro M, Pritsch M, du Plessis JD, Shock J, Schwienhorst-Stich EM, et al. Understanding the widespread use of veterinary ivermectin for Chagas disease, underlying factors and implications for the COVID-19 pandemic: a convergent mixed-methods study. BMJ Open. 2022 Sep 17;12(9):e058572.

2. Flottorp SA, Oxman AD, Krause J, Musila NR, Wensing M, Godycki-Cwirko M, et al. A checklist for identifying determinants of practice: A systematic review and synthesis of frameworks and taxonomies of factors that prevent or enable improvements in healthcare professional practice. Vol. 8, Implementation Science. 2013.

3. Damschroder LJ, Aron DC, Keith RE, Kirsh SR, Alexander JA, Lowery JC. Fostering implementation of health services research findings into practice: A consolidated framework for advancing implementation science. Implementation Science. 2009;4(1).
