# Supplementary material for: Practice determinants for adherence to the Guide for the Comprehensive Clinical Care of Dengue Patients, Urabá (Colombia). A multifaceted approach to implementation research
Source: PLoS Negl Trop Dis. 2024 Aug 15;18(8):e0012361. doi: 10.1371/journal.pntd.0012361 (PMC11349210; doi:10.1371/journal.pntd.0012361)
Supplement: S2 Appendix — Table A. Consolidated criteria for reporting qualitative studies (COREQ): 32-item checklist. Table B. STROBE Statement—checklist of items that should be included in reports of observational studies. (DOC) [file pntd.0012361.s004.doc]

**Annex 2: Supplementary Material S2**

1. **Table A Consolidated criteria for reporting qualitative studies (COREQ): 32-item checklist**

Developed from:

Tong A, Sainsbury P, Craig J. Consolidated criteria for reporting qualitative research (COREQ): a 32-item checklist for interviews and focus groups. *International Journal for Quality in Health Care*. 2007. 19 (6): pp. 349 – 357

| **No. Item** | **Guide questions / description** | **Reported on Page #** |
| --- | --- | --- |
| **Domain A: Research team and reﬂexivity** |  |  |
| *Personal Characteristics* |  |  |
| 1. Interviewer/facilitator | Which author/s conducted the interview or focus group? | Page 1  Manuscript |
| 2. Credentials | What were the researcher’s credentials? E.g., PhD, MD | Title page* |
| 3. Occupation | What was the researcher’s occupation at the time of the study? | Title page* |
| 4. Gender | Was the researcher male or female? | Title page* |
| 5. Experience and training | What training experience did the researcher have? | Title page* |
| *Relationship with participants* |  |  |
| 6. Relationship established | Was a relationship established prior to study commencement? | Manuscript |
| 7. Participant knowledge of the interviewer | What did the participants know about the researcher? e.g., personal goals, reasons for doing the research | Manuscript |
| 8. Interviewer characteristics | What characteristics were reported about the interviewer/facilitator? e.g., Biases, assumptions, reasons and interests in the research topic | Manuscript |
| **Domain B: study design** |  |  |
| *Theoretical framework* |  |  |
| 9. Methodological orientation and Theory | What methodological orientation was stated to underpin the study? e.g., grounded theory, discourse analysis, ethnography, phenomenology, content analysis | Title page  Page 1 (abstract) & 3 (main document) |
| *Participant selection* |  |  |
| 10. Sampling | How were participants selected? e.g., purposive, convenience, consecutive, snowball | Page 4 & 5  Manuscript |
| 11. Method of approach | How were participants approached? e.g., face-to-face, telephone, mail, email | Page 4 & 5  Manuscript |
| 12. Sample size | How many participants were in the study? | Page 4 & 5  Manuscript |
| 13. Non-participation | How many people refused to participate or dropped out? Reasons? | Page 4  Manuscript.  (A purposive sample of nurses was initially recruited through social media (i.e., Facebook). Then, the snowballing sampling method was applied to recruit potential participants). Therefore, nurses only who expressed their willingness to participate in this study were included. Therefore, no non-participation |
| *Setting* |  |  |
| 14. Setting of data collection | Where was the data collected? e.g., home, clinic, workplace | Page 4  Manuscript  . |
| 15. Presence of non-participants | Was anyone else present besides the participants and researchers? | Page 4 & 5  Manuscript  Only the first researcher and interviewee participated.  In-depth telephone interviews and/or virtual meetings were conducted by the first researcher and the qualitative research expert, during June 2020, using an interview guide developed by the research team based on the literature and aim of this study. The telephone an virtual methods helped to collect data during the curfew period with travel restriction. After identifying the potential participants, we distributed written information sheets and consent forms via electronic media (i.e., email and WhatsApp). Possible time for both parties was set.”. |
| 16. Sample description | What are the important characteristics of the sample? e.g., demographic data, date | Page 4, & 5, including Table 2  Manuscript |
| *Data collection* |  |  |
| 17. Interview guide | Were questions, prompts, guides provided by the authors? Was it pilot tested? | Page 4 & 5, including table 1.  Manuscript |
| 18. Repeat interviews | Were repeat interviews carried out? If so, how many? | No |
| 19. Audio/visual recording | Did the research use audio or visual recording to collect the data? | Page 4 & 5  Manuscript |
| 20. Field notes | Were ﬁeld notes made during and/or after the interview or focus group? | Reflective notes were taken following the interviews |
| 21. Duration | What was the duration of the interviews or focus group? | Page 5  Manuscript |
| 22. Data saturation | Was data saturation discussed? | Page 4  Manuscript |
| 23. Transcripts returned | Were transcripts returned to participants for comment and/or correction? | Page 5  Manuscript |
| **Domain C: analysis and ﬁndings** |  |  |
| *Data analysis* |  |  |
| 24. Number of data coders | How many data coders coded the data? | Page 5  Manuscript |
| 25. Description of the coding tree | Did authors provide a description of the coding tree? | Themes and sub-themes were provided in a table.  Table 3, Page 6 & 7. Manuscript |
| 26. Derivation of themes | Were themes identiﬁed in advance or derived from the data? | Derived from data.  Page 5 & 6  Manuscript |
| 27. Software | What software, if applicable, was used to manage the data? | Data were manually manipulated using a word processor. |
| 28. Participant checking | Did participants provide feedback on the ﬁndings? | Page 6  Manuscript |
| *Reporting* |  |  |
| 29. Quotations presented | Were participant quotations presented to illustrate the themes/ﬁndings? Was each quotation identiﬁed? e.g., participant number | Page 6 to 11  Manuscript  Annex 4, Supplementary Material S4 |
| 30. Consistency of data and ﬁndings | Was there consistency between the data presented and the ﬁndings? | Yes, there was.  Pages 6 to 11  Manuscript |
| 31. Clarity of major themes | Were major themes clearly presented in the ﬁndings? | Yes, they were.  From page 6-11 & Table 3 in pages 6 & 7  Manuscript |
| 32. Clarity of minor themes | Is there a description of diverse cases or discussion of minor themes? | Discussion of major and minor themes  From page 11-13 and Table 3 in pages 6 & 7.  Manuscript |

1. **Table B. STROBE Statement—checklist of items that should be included in reports of observational studies**

|  | Item No | Recommendation |
| --- | --- | --- |
| **Title and abstract** | 1 | (*a*) Indicate the study’s design with a commonly used term in the title or the abstract |
|  |  | (*b*) Provide in the abstract an informative and balanced summary of what was done and what was found |
| Introduction | | |
| Background/rationale | 2 | Explain the scientific background and rationale for the investigation being reported |
| Objectives | 3 | State specific objectives, including any pre-specified hypotheses |
| Methods | | |
| Study design | 4 | Present key elements of study design early in the paper |
| Setting | 5 | Describe the setting, locations, and relevant dates, including periods of recruitment, exposure, follow-up, and data collection |
| Participants | 6 | (*a*) *Cohort study*-Give the eligibility criteria, and the sources and methods of selection of participants. Describe methods of follow-up  *Case-control study*-Give the eligibility criteria, and the sources and methods of case ascertainment and control selection. Give the rationale for the choice of cases and controls  *Cross-sectional study*—Give the eligibility criteria, and the sources and methods of selection of participants |
|  |  | (*b*) *Cohort study*-For matched studies, give matching criteria and number of exposed and unexposed  *Case-control study*-For matched studies, give matching criteria and the number of controls per case |
| Variables | 7 | Clearly define all outcomes, exposures, predictors, potential confounders, and effect modifiers. Give diagnostic criteria, if applicable |
| Data sources/ measurement | 8* | For each variable of interest, give sources of data and details of methods of assessment (measurement). Describe comparability of assessment methods if there is more than one group |
| Bias | 9 | Describe any efforts to address potential sources of bias |
| Study size | 10 | Explain how the study size was arrived at |
| Quantitative variables | 11 | Explain how quantitative variables were handled in the analyses. If applicable, describe which groupings were chosen and why |
| Statistical methods | 12 | (*a*) Describe all statistical methods, including those used to control for confounding variables |
|  |  | (*b*) Describe any methods used to examine subgroups and interactions |
|  |  | (*c*) Explain how missing data were addressed |
|  |  | (*d*) *Cohort study -*If applicable, explain how loss to follow-up was addressed  *Case-control study* -If applicable, explain how matching of cases and controls was addressed  *Cross-sectional study* -If applicable, describe analytical methods taking account of sampling strategy |
|  |  | (*e*) Describe any sensitivity analyses |

| Results | | |
| --- | --- | --- |
| Participants | 13* | (a) Report numbers of individuals at each stage of study e.g., numbers potentially eligible, examined for eligibility, confirmed eligible, included in the study, completing follow-up, and analysed |
|  |  | (b) Give reasons for non-participation at each stage |
|  |  | (c) Consider the use of a flow diagram |
| Descriptive data | 14* | (a) Give characteristics of study participants (e.g., demographic, clinical, social) and information on exposures and potential confounders |
|  |  | (b) Indicate the number of participants with missing data for each variable of interest |
|  |  | (c) *Cohort study*—Summarise follow-up time (e.g., average and total amount) |
| Outcome data | 15* | *Cohort study*—Report numbers of outcome events or summary measures over time |
|  |  | *Case-control study-*Report numbers in each exposure category, or summary measures of exposure |
|  |  | *Cross-sectional study -* Report numbers of outcome events or summary measures |
| Main results | 16 | (*a*) Give unadjusted estimates and, if applicable, confounder-adjusted estimates and their precision (e.g., 95% confidence interval). Make clear which confounders were adjusted for and why they were included |
|  |  | (*b*) Report category boundaries when continuous variables were categorized |
|  |  | (*c*) If relevant, consider translating estimates of relative risk into absolute risk for a meaningful time period |
| Other analyses | 17 | Report other analyses done—e.g., analyses of subgroups and interactions, and sensitivity analyses |
| Discussion | | |
| Key results | 18 | Summarise key results with reference to study objectives |
| Limitations | 19 | Discuss limitations of the study, taking into account sources of potential bias or imprecision. Discuss both direction and magnitude of any potential bias |
| Interpretation | 20 | Give a cautious overall interpretation of results considering objectives, limitations, multiplicity of analyses, results from similar studies, and other relevant evidence |
| Generalisability | 21 | Discuss the generalisability (external validity) of the study results |
| Other information | | |
| Funding | 22 | Give the source of funding and the role of the funders for the present study and, if applicable, for the original study on which the present article is based |

*Give information separately for cases and controls in case-control studies and, if applicable, for exposed and unexposed groups in cohort and cross-sectional studies.

**Note:** An Explanation and Elaboration article discusses each checklist item and gives methodological background and published examples of transparent reporting. The STROBE checklist is best used in conjunction with this article (freely available on the Web sites of PLoS Medicine at http://www.plosmedicine.org/, Annals of Internal Medicine at http://www.annals.org/, and Epidemiology at http://www.epidem.com/). Information on the STROBE Initiative is available at www.strobe-statement.org.
