# Supplementary material for: Practice determinants for adherence to the Guide for the Comprehensive Clinical Care of Dengue Patients, Urabá (Colombia). A multifaceted approach to implementation research
Source: PLoS Negl Trop Dis. 2024 Aug 15;18(8):e0012361. doi: 10.1371/journal.pntd.0012361 (PMC11349210; doi:10.1371/journal.pntd.0012361)
Supplement: S3 Appendix — Table A. Statistical analysis of the perception of the determinants of adherence to the GACIPD according to each domain. Table B. Statistical analysis of the perception of the determinants of adherence to the GACIPD, according to time of professional experience. Urabá 2021 online survey. Table C. Statistical analysis of the perception of the determinants of adherence to the GACIPD, according to time of professional experience. Urabá 2021 online survey. (DOCX) [file pntd.0012361.s005.docx]

**Supplementary Material S3**

**Table A: Statistical analysis of the perception of the determinants of adherence to the GACIPD according to each domain. Online questionnaire Urabá 2021 n=136**

| **SUBJECTS** | **18-42 LOW 43-66 MEDIUM 67-90 HIGH** | **6-14 LOW 15-21 MEDIUM 22-30 HIGH** | **5-11 LOW 12-18 MEDIUM 19-25 HIGH** | **2-4 LOW 5-6 MEDIUM 7-10 HIGH** | **7-16 LOW 17-25 MEDIUM 26-35 HIGH** | **4-9 LOW 10-15 MEDIUM 16-20 HIGH** | **2-4 LOW 5-6 MEDIUM 7-10 HIGH** | **44-103 LOW 104-162 MEDIUM 163-220 HIGH** |
| --- | --- | --- | --- | --- | --- | --- | --- | --- |
|  | DA | DB | DC | DD | DE | DF | DG | **TOTAL** |
| 1 | 62 | 17 | 18 | 7 | 28 | 15 | 9 | 156 |
| 2 | 42 | 20 | 11 | 7 | 19 | 8 | 6 | 113 |
| 3 | 28 | 11 | 6 | 2 | 12 | 5 | 2 | 66 |
| 4 | 42 | 23 | 15 | 7 | 26 | 12 | 7 | 132 |
| 5 | 66 | 21 | 8 | 5 | 33 | 19 | 2 | 154 |
| 6 | 68 | 23 | 17 | 6 | 10 | 4 | 5 | 133 |
| 7 | 73 | 12 | 15 | 6 | 15 | 10 | 2 | 133 |
| 8 | 72 | 16 | 17 | 5 | 26 | 14 | 5 | 155 |
| 9 | 63 | 14 | 16 | 7 | 26 | 14 | 6 | 146 |
| 10 | 73 | 22 | 20 | 7 | 28 | 16 | 8 | 174 |
| 11 | 71 | 19 | 16 | 5 | 26 | 14 | 6 | 157 |
| 12 | 54 | 6 | 12 | 6 | 7 | 4 | 2 | 91 |
| 13 | 76 | 10 | 18 | 8 | 22 | 10 | 8 | 152 |
| 14 | 81 | 18 | 19 | 5 | 20 | 13 | 8 | 164 |
| 15 | 22 | 6 | 5 | 2 | 7 | 4 | 2 | 48 |
| 16 | 67 | 12 | 17 | 5 | 12 | 4 | 2 | 119 |
| 17 | 59 | 11 | 14 | 5 | 16 | 4 | 5 | 114 |
| 18 | 64 | 15 | 15 | 6 | 22 | 12 | 3 | 137 |
| 19 | 69 | 24 | 20 | 8 | 28 | 16 | 8 | 173 |
| 20 | 66 | 11 | 21 | 3 | 22 | 9 | 6 | 138 |
| 21 | 75 | 26 | 20 | 9 | 27 | 16 | 6 | 179 |
| 22 | 69 | 20 | 18 | 6 | 24 | 15 | 5 | 157 |
| 23 | 70 | 19 | 19 | 6 | 24 | 16 | 8 | 162 |
| 24 | 75 | 24 | 18 | 8 | 26 | 16 | 8 | 175 |
| 25 | 63 | 13 | 16 | 4 | 25 | 12 | 2 | 135 |
| 26 | 56 | 16 | 18 | 5 | 26 | 15 | 8 | 144 |
| 27 | 20 | 6 | 5 | 2 | 7 | 4 | 2 | 46 |
| 28 | 63 | 6 | 24 | 5 | 13 | 7 | 4 | 122 |
| 29 | 71 | 12 | 19 | 7 | 22 | 13 | 8 | 152 |
| 30 | 42 | 6 | 5 | 2 | 7 | 4 | 2 | 68 |
| 31 | 72 | 24 | 20 | 8 | 28 | 16 | 8 | 176 |
| 32 | 72 | 24 | 20 | 5 | 21 | 15 | 4 | 161 |
| 33 | 67 | 23 | 20 | 8 | 28 | 16 | 8 | 170 |
| 34 | 66 | 22 | 17 | 5 | 28 | 11 | 5 | 154 |
| 35 | 70 | 19 | 18 | 6 | 27 | 14 | 6 | 160 |
| 36 | 69 | 11 | 13 | 6 | 22 | 12 | 5 | 138 |
| 37 | 73 | 18 | 19 | 7 | 27 | 14 | 8 | 166 |
| 38 | 83 | 22 | 19 | 8 | 29 | 16 | 9 | 186 |
| 39 | 65 | 16 | 18 | 6 | 19 | 9 | 6 | 139 |
| 40 | 80 | 24 | 18 | 5 | 28 | 15 | 6 | 176 |
| 41 | 82 | 16 | 20 | 5 | 23 | 7 | 4 | 157 |
| 42 | 72 | 22 | 18 | 6 | 26 | 16 | 6 | 166 |
| 43 | 26 | 12 | 8 | 2 | 16 | 4 | 6 | 74 |
| 44 | 69 | 16 | 21 | 5 | 32 | 17 | 6 | 166 |
| 45 | 74 | 17 | 19 | 5 | 29 | 16 | 5 | 165 |
| 46 | 72 | 24 | 20 | 8 | 28 | 16 | 8 | 176 |
| 47 | 63 | 16 | 17 | 5 | 22 | 16 | 9 | 148 |
| 48 | 45 | 10 | 18 | 8 | 22 | 6 | 8 | 117 |
| 49 | 68 | 21 | 20 | 8 | 33 | 18 | 8 | 176 |
| 50 | 69 | 22 | 18 | 6 | 25 | 17 | 6 | 163 |
| 51 | 75 | 22 | 22 | 6 | 28 | 14 | 4 | 171 |
| 52 | 84 | 26 | 16 | 9 | 31 | 17 | 9 | 192 |
| 53 | 79 | 26 | 22 | 6 | 27 | 15 | 7 | 182 |
| 54 | 69 | 22 | 20 | 4 | 25 | 16 | 5 | 161 |
| 55 | 65 | 12 | 18 | 5 | 19 | 10 | 6 | 135 |
| 56 | 58 | 6 | 5 | 2 | 7 | 4 | 2 | 84 |
| 57 | 36 | 9 | 8 | 5 | 15 | 4 | 2 | 79 |
| 58 | 65 | 17 | 18 | 6 | 25 | 12 | 6 | 149 |
| 59 | 64 | 21 | 18 | 6 | 21 | 12 | 7 | 149 |
| 60 | 73 | 21 | 16 | 8 | 27 | 12 | 7 | 164 |
| 61 | 83 | 30 | 25 | 10 | 31 | 16 | 6 | 201 |
| 62 | 62 | 17 | 14 | 7 | 21 | 15 | 6 | 142 |
| 63 | 58 | 15 | 20 | 5 | 19 | 10 | 5 | 132 |
| 64 | 67 | 17 | 19 | 8 | 25 | 13 | 7 | 156 |
| 65 | 72 | 24 | 20 | 4 | 28 | 17 | 8 | 173 |
| 66 | 69 | 22 | 20 | 8 | 27 | 14 | 4 | 164 |
| 67 | 75 | 19 | 16 | 6 | 22 | 14 | 6 | 158 |
| 68 | 71 | 23 | 19 | 8 | 28 | 14 | 8 | 171 |
| 69 | 86 | 22 | 18 | 7 | 28 | 15 | 3 | 179 |
| 70 | 88 | 24 | 20 | 8 | 30 | 16 | 7 | 193 |
| 71 | 81 | 26 | 21 | 8 | 25 | 13 | 4 | 178 |
| 72 | 69 | 15 | 17 | 5 | 21 | 10 | 2 | 139 |
| 73 | 74 | 16 | 18 | 6 | 26 | 11 | 6 | 157 |
| 74 | 78 | 22 | 21 | 8 | 26 | 16 | 6 | 177 |
| 75 | 60 | 7 | 12 | 6 | 20 | 7 | 6 | 118 |
| 76 | 70 | 20 | 20 | 5 | 25 | 16 | 4 | 160 |
| 77 | 72 | 24 | 20 | 8 | 28 | 16 | 8 | 176 |
| 78 | 69 | 22 | 19 | 7 | 25 | 14 | 7 | 163 |
| 79 | 57 | 15 | 17 | 2 | 16 | 4 | 2 | 113 |
| 80 | 72 | 24 | 22 | 8 | 28 | 16 | 8 | 178 |
| 81 | 90 | 27 | 20 | 8 | 35 | 18 | 8 | 206 |
| 82 | 62 | 19 | 19 | 5 | 23 | 13 | 6 | 147 |
| 83 | 84 | 21 | 22 | 7 | 31 | 19 | 4 | 188 |
| 84 | 70 | 22 | 19 | 5 | 28 | 14 | 5 | 163 |
| 85 | 56 | 15 | 15 | 3 | 16 | 5 | 7 | 117 |
| 86 | 76 | 23 | 20 | 5 | 31 | 13 | 6 | 174 |
| 87 | 69 | 13 | 17 | 6 | 28 | 16 | 4 | 153 |
| 88 | 41 | 12 | 18 | 5 | 23 | 9 | 6 | 114 |
| 89 | 71 | 20 | 18 | 5 | 21 | 15 | 4 | 154 |
| 90 | 39 | 22 | 20 | 8 | 29 | 16 | 8 | 142 |
| 91 | 64 | 15 | 19 | 9 | 33 | 20 | 10 | 170 |
| 92 | 52 | 18 | 15 | 6 | 21 | 12 | 6 | 130 |
| 93 | 87 | 23 | 18 | 6 | 26 | 16 | 6 | 182 |
| 94 | 77 | 29 | 23 | 8 | 31 | 19 | 7 | 194 |
| 95 | 73 | 24 | 20 | 8 | 28 | 16 | 8 | 177 |
| 96 | 80 | 25 | 21 | 8 | 30 | 16 | 6 | 186 |
| 97 | 79 | 26 | 20 | 8 | 28 | 16 | 8 | 185 |
| 98 | 77 | 30 | 23 | 8 | 30 | 16 | 9 | 193 |
| 99 | 66 | 20 | 20 | 6 | 23 | 13 | 6 | 154 |
| 100 | 84 | 27 | 25 | 8 | 31 | 17 | 9 | 201 |
| 101 | 66 | 16 | 14 | 6 | 18 | 12 | 4 | 136 |
| 102 | 70 | 18 | 18 | 7 | 24 | 14 | 6 | 157 |
| 103 | 72 | 18 | 15 | 6 | 21 | 12 | 6 | 150 |
| 104 | 48 | 16 | 20 | 7 | 24 | 13 | 5 | 133 |
| 105 | 74 | 21 | 17 | 5 | 28 | 10 | 2 | 157 |
| 106 | 86 | 27 | 20 | 7 | 25 | 16 | 2 | 183 |
| 107 | 55 | 18 | 23 | 4 | 24 | 16 | 5 | 145 |
| 108 | 76 | 13 | 19 | 6 | 23 | 12 | 7 | 156 |
| 109 | 89 | 27 | 21 | 6 | 32 | 20 | 8 | 203 |
| 110 | 72 | 17 | 20 | 7 | 22 | 13 | 8 | 159 |
| 111 | 71 | 22 | 19 | 6 | 26 | 14 | 8 | 166 |
| 112 | 66 | 21 | 16 | 8 | 29 | 14 | 6 | 160 |
| 113 | 53 | 11 | 15 | 6 | 28 | 16 | 5 | 134 |
| 114 | 75 | 12 | 17 | 5 | 16 | 8 | 5 | 138 |
| 115 | 79 | 12 | 17 | 5 | 16 | 8 | 5 | 142 |
| 116 | 71 | 21 | 21 | 8 | 23 | 11 | 10 | 165 |
| 117 | 46 | 16 | 14 | 7 | 21 | 7 | 9 | 120 |
| 118 | 70 | 22 | 18 | 6 | 28 | 15 | 9 | 168 |
| 119 | 57 | 17 | 20 | 6 | 26 | 17 | 4 | 147 |
| 120 | 64 | 9 | 19 | 5 | 24 | 15 | 8 | 144 |
| 121 | 58 | 20 | 18 | 5 | 26 | 13 | 8 | 148 |
| 122 | 51 | 12 | 21 | 6 | 22 | 12 | 6 | 130 |
| 123 | 53 | 23 | 22 | 2 | 25 | 12 | 6 | 143 |
| 124 | 77 | 22 | 17 | 5 | 28 | 16 | 8 | 173 |
| 125 | 39 | 6 | 14 | 2 | 16 | 7 | 5 | 89 |
| 126 | 42 | 18 | 11 | 5 | 16 | 13 | 5 | 110 |
| 127 | 73 | 24 | 20 | 8 | 28 | 16 | 8 | 177 |
| 128 | 72 | 18 | 18 | 7 | 29 | 15 | 6 | 165 |
| 129 | 88 | 24 | 20 | 8 | 30 | 16 | 7 | 193 |
| 130 | 28 | 6 | 5 | 2 | 7 | 4 | 2 | 54 |
| 131 | 87 | 22 | 22 | 6 | 27 | 16 | 6 | 186 |
| 132 | 63 | 25 | 15 | 6 | 28 | 13 | 7 | 157 |
| 133 | 64 | 19 | 15 | 6 | 25 | 16 | 8 | 153 |
| 134 | 80 | 11 | 15 | 4 | 26 | 14 | 6 | 156 |
| 135 | 71 | 16 | 15 | 7 | 26 | 15 | 4 | 154 |
| 136 | 85 | 16 | 19 | 6 | 33 | 14 | 7 | 180 |
| Average | 66,7 | 18,3 | 17,5 | 6,0 | 24,0 | 12,9 | 5,9 | 151,4 |

* DA: Determinants of guideline use; DB: Individual health professional factors; DC: Patient factors; DD: Professional interactions factors; DE: Incentive and resource factors; DF: Capacity for organizational change; DG: Social, political, and legal factors. **Score range: Minimum and maximum score for each domain stratified into categories.

**Table B: Statistical analysis of the perception of the determinants of adherence to the GACIPD, according to time of professional experience. Urabá 2021 online survey.**

| **DOMAIN** | **SCORE RANGE** | **STATISTIC** | **1 year or less** | **2 or 4 years** | **5 or 9 years** | **10 year or more** | **Chi^2^** | **(P- Value)** |
| --- | --- | --- | --- | --- | --- | --- | --- | --- |
| **D A** | 18-42 LOW 43-66 MEDIUM 67-90 HIGH | Average | 68 | 67 | 67,2 | 67,2 | 145,4 | 0.523 |
|  |  | SD | 13 | 14 | 13,6 | 13,6 |  |  |
| **D B** | 6-14 LOW 15-21 MEDIUM 22-30 HIGH | Average | 19,2 | 18,4 | 18,5 | 18,4 | 66,7 | 0.457 |
|  |  | SD | 5,5 | 5,8 | 5,9 | 5,8 |  |  |
| **D C** | 5-11 LOW 12-18 MEDIUM 19-25 HIGH | Average | 18,2 | 17,6 | 17,7 | 17,7 | 49,9 | 0.519 |
|  |  | SD | 3,5 | 4,0 | 4,1 | 3,9 |  |  |
| **D D** | 2-4 LOW 5-6 MEDIUM 7-10 HIGH | Average | 6,2 | 6,0 | 6,1 | 6,0 | 24,8 | 0.415 |
|  |  | SD | 1,7 | 1,8 | 1,8 | 1,7 |  |  |
| **D E** | 7-16 LOW 17-25 MEDIUM 26-35 HIGH | Average | 24,9 | 23,9 | 23,9 | 24,1 | 71,4 | 0.304 |
|  |  | SD | 5,2 | 6,0 | 6,2 | 6,0 |  |  |
| **D F** | 4-9 LOW 10-15 MEDIUM 16-20 HIGH | Average | 13,4 | 12,9 | 12,8 | 13,0 | 47,1 | 0.508 |
|  |  | SD | 3,8 | 4,1 | 4,2 | 4,0 |  |  |
| **D G** | 2-4 LOW 5-6 MEDIUM 7-10 HIGH | Average | 6,2 | 5,9 | 5,7 | 5,9 | 26,2 | 0.344 |
|  |  | SD | 1,9 | 2,1 | 2,1 | 2,0 |  |  |
| **TOTAL** | 44-103 LOW 104-162 MEDIUM 163-220 HIGH | Average | 156,1 | 151,1 | 151,9 | 152,2 | 229,2 | 0.256 |
|  |  | SD | 28,6 | 31,5 | 31,7 | 30,7 |  |  |

* DA: Determinants of guideline use; DB: Individual health professional factors; DC: Patient factors; DD: Professional interactions factors; DE: Incentive and resource factors; DF: Capacity for organizational change; DG: Social, political and legal factors. **Score range: Minimum and maximum score for each domain stratified into categories; ***Chi^2^: Chi^2^: Statistical association test.

**Table C: Statistical analysis of the perception of the determinants of adherence to the GACIPD, according to time of professional experience. Urabá 2021 online survey.**

| **DOMAIN*** | **Score Range** | **Total** | | **1 year or less** | | **2 to 4 years** | | **5 to 9 years** | | **10 years or more** | | **Mean** | **KW**** |
| --- | --- | --- | --- | --- | --- | --- | --- | --- | --- | --- | --- | --- | --- |
|  |  | **n** | **%** | **n=18** | **%** | **n=39** | **%** | **n=23** | **%** | **n=56** | **%** | **(SD)** | **(p value)** |
| **D A** | **18-42 LOW** | 13 | 9,6 | 1 | 5,6 | 4 | 10,3 | 3 | 13,0 | 5 | 8,9 | 66,7 (14,0) | 0,37 (0,944) |
|  | **43-66 MEDIUM** | 40 | 29,4 | 6 | 33,3 | 11 | 28,2 | 6 | 26,1 | 17 | 30,4 |  |  |
|  | **67-90 HIGH** | 83 | 61,0 | 11 | 61,1 | 24 | 61,5 | 14 | 60,9 | 34 | 60,7 |  |  |
| **D B** | **6-14 LOW** | 32 | 23,5 | 2 | 11,1 | 8 | 20,5 | 8 | 34,8 | 14 | 25,0 | 18.31 (5,8) | 2,7 (0,430) |
|  | **15-21 MEDIUM** | 53 | 39,0 | 6 | 33,3 | 17 | 43,6 | 7 | 30,4 | 23 | 41,1 |  |  |
|  | **22-30 HIGH** | 51 | 37,5 | 10 | 55,6 | 14 | 35,9 | 8 | 34,8 | 19 | 33,9 |  |  |
| **D C** | **5-11 LOW** | 11 | 8,1 | 1 | 5,6 | 2 | 5,1 | 3 | 13,0 | 5 | 8,9 | 17,5 (4,0) | 1,32 (0,723) |
|  | **12-18 MEDIUM** | 61 | 44,9 | 8 | 44,4 | 18 | 46,2 | 9 | 39,1 | 26 | 46,4 |  |  |
|  | **19-25 HIGH** | 64 | 47,1 | 9 | 50,0 | 19 | 48,7 | 11 | 47,8 | 25 | 44,6 |  |  |
| **D D** | **2-4 LOW** | 17 | 12,5 | 2 | 11,1 | 2 | 5,1 | 6 | 26,1 | 7 | 12,5 | 7,2 (2,2) | 1,33 (0,722) |
|  | **5-6 MEDIUM** | 67 | 49,3 | 8 | 44,4 | 22 | 56,4 | 8 | 34,8 | 29 | 51,79 |  |  |
|  | **7-10 HIGH** | 52 | 38,2 | 8 | 44,4 | 15 | 38,5 | 9 | 39,1 | 20 | 35,71 |  |  |
| **D E** | **7-16 LOW** | 20 | 14,7 | 8 | 44,4 | 5 | 12,8 | 4 | 17,4 | 9 | 16,07 | 28,7 (6,9) | 1,6 (0,640) |
|  | **17-25 MEDIUM** | 48 | 35,3 | 5 | 27,8 | 17 | 43,6 | 9 | 39,1 | 17 | 30,36 |  |  |
|  | **26-35 HIGH** | 68 | 50,0 | 11 | 61,1 | 17 | 43,6 | 10 | 43,5 | 30 | 53,57 |  |  |
| **D F** | **4-9 LOW** | 26 | 19,1 | 4 | 22,2 | 6 | 15,4 | 5 | 21,7 | 11 | 19,64 | 12,6 (4,1) | 0,89 (0,840) |
|  | **10-15 MEDIUM** | 63 | 46,3 | 5 | 27,8 | 24 | 61,5 | 10 | 43,5 | 24 | 42,86 |  |  |
|  | **16-20 HIGH** | 47 | 34,6 | 9 | 50,0 | 9 | 23,1 | 8 | 34,8 | 21 | 37,5 |  |  |
| **D G** | **2-4 LOW** | 31 | 22,8 | 3 | 16,7 | 8 | 20,5 | 8 | 34,8 | 12 | 21,43 | 7,1 (2,4) | 4,01 (0,259) |
|  | **5-6 MEDIUM** | 52 | 38,2 | 7 | 38,9 | 18 | 46,2 | 9 | 39,1 | 18 | 32,14 |  |  |
|  | **7-10 HIGH** | 53 | 39,0 | 8 | 44,4 | 13 | 33,3 | 6 | 26,1 | 26 | 46,43 |  |  |
| **TOTAL** | **44-103 LOW** | 10 | 7,4 | 1 | 5,6 | 1 | 2,6 | 3 | 13,0 | 5 | 8,9 | 131,4 (31,4) | 1,92 (0,587) |
|  | **104-162 MEDIUM** | 72 | 52,9 | 7 | 38,9 | 27 | 69,2 | 10 | 43,5 | 28 | 50,0 |  |  |
|  | **163-220 HIGH** | 54 | 39,7 | 10 | 55,6 | 11 | 28,2 | 10 | 43,5 | 23 | 41,1 |  |  |

* DA: Determinants of guideline use; DB: Individual health professional factors; DC: Patient factors; DD: Professional interaction factors; DE: Incentive and resource factors; DF: Capacity for organizational change; DG: Social, political, and legal factors. **Score range: Minimum and maximum score for each domain stratified into categories; **Kruskal Wallis: Statistical association test of GACIPD adherence determinants, according to years of experience. Online Questionnaire Urabá 2021
