# Supplementary material for: Practice determinants for adherence to the Guide for the Comprehensive Clinical Care of Dengue Patients, Urabá (Colombia). A multifaceted approach to implementation research
Source: PLoS Negl Trop Dis. 2024 Aug 15;18(8):e0012361. doi: 10.1371/journal.pntd.0012361 (PMC11349210; doi:10.1371/journal.pntd.0012361)
Supplement: S4 Appendix — Table A. Results of the semi-structured interviews and focus groups. Table B. Results of focus groups. Table C. Results of the semi-structured interviews. (DOCX) [file pntd.0012361.s006.docx]

**Supplementary Material S4**

**Table A: Results of the semi-structured interviews and focus groups**

| Word | Domain | Determinant | Subject | Barriers | Facilitators |
| --- | --- | --- | --- | --- | --- |
| A | Guideline Factors: GACIPD | How GACIPD  was developed | To be in line with current developments in GPC design | "It is unknown that a guide is elaborated by experts and validated from evidence-based medicine, a lack of knowledge that comes from the undergraduate training programs" (**PSSI**:7).  "The guide is not very clear when a patient has renal failure, when a patient is diabetic, when a patient is hypertensive, when a patient has thousands of infarctions and has dengue, and the guide is not clear on that, or when it comes with co-infections, leptospirosis, malaria" (**FGP**:7). | "the guidance is very clear in saying what to do with a patient who only has dengue," **FGP**:7). |
|  |  | To be able to work with GACIPD | To be in line with the current form of consultation in GPC. | It is also mentioned that the guide is outdated "The Dengue Guide is outdated. It is a guide that is not in line with current regulations, with the regulatory and administrative process"  (**PSSI**.1,3,4,5,6). | "It seems to me that the guide is very important to analyze not only the clinical context, but also the social context, psychology", (**FGP**:2). |
|  |  |  | Experimenting/practicing with GPCs | "I have no knowledge about the guide, but I know something about dengue" (**FGP**:1), "we don't read the guide like that" (**FGP**:2) "regarding the guide is that I have realized that nobody knows it, but they know what they  talk about here" (**FGP**:10). | In any case, it is worth mentioning that this is the domain of the guide that deserves the most comments, with a consensus on its usefulness, easy access and as a support in medical practice for decision making (**PSSI**. 1, 2,3,4,5,6,7).  "I think it is a clear, concise, precise guide." (**FGP**:1). |
|  |  | Behavior required for the use of the GACIPD | Incorporate in local guidelines | "The current guide is 11 years old, very academic, but with big gaps and there have been regulatory and (PIC, institutional environment, work etc., integrated management, RIAS, Rapid tests, resolution 3280) administrative changes that are not reflected in these guides. It is a guide that is not in line with current regulations, with the regulatory and administrative process. There is a great disadvantage in not updating the guidelines on a permanent basis". (**PSSI**: 1) |  |
|  |  |  |  | "Basically there was not a socialization as such that guides the protocol" (**FGP**:10), "In the year before last the health secretariat gave us a training on dengue, but not for years back in the year we have not had training on dengue" (**FGP**:8), "a guideline as such does not exist, I was told when you have a suspected patient what you have to do is based on what you already have as knowledge of the pathology"  (**FGP**:10). |  |
| B | Individual health professional factors | Knowledge and skills of health teams. | Professionals are not familiar with the subject | "Generally now there are many new doctors, so doctors rotate a lot, today we have one, tomorrow we have another...trained, I say, by certain pathologies to which they adhere, so that is very difficult" (**FGP**: 8). | they can be resolved through ICT strategies: videos, virtual spaces, podcasts, listening to what people want to be explained, through images and listening" (**PSSI**: 6). ICTs: Information and communication technologies. |
|  |  |  |  | "Well, how important is it from the laboratory's point of view that they do the test that is much more sensitive and that we do not have it in the institution, I understand that it is a limitation for us as physicians" (**PSSI**: 3). |  |
|  |  | Knowledge, beliefs and attitudes of health teams. | Attention to individual needs and competencies | “From undergraduate training there is not much emphasis for knowledge of the guide" (**PSSI**: 6).  "There are students already graduated from the undergraduate program who come with many gaps in their knowledge about the dengue guide"; (**PSSI**:5).  "In medical schools they are not made known, they are only known in practice when faced with a patient." (**PSSI:** 4) |  |
|  |  |  |  | "A guideline as such does not exist, I was told that when you have a suspicious patient, what you have to do is based on what you already know about the pathology" (**FGP**:10). |  |
|  |  |  |  | "So I think that if we are going to look at this guide, it lacks, it lacks patients with comorbidities and if it says a little bit over there it is very brief and we know and understand that they are more complicated, which are precisely those who have comorbidities." (**FGP**:4) |  |
|  |  | Professional behavior of health teams | Self-confidence in one's own ability | "It is necessary to demystify that guidelines take away autonomy, in other fields such as nursing; I have seen it in medicine: ah, but then where is my knowledge, my autonomy to treat patients, as I believe? if we manage to break that scheme in the feeling, we can overcome the saying, is that the doctor, nurse,  nurse or microbiologist is autonomous." (**PSSI:**1). |  |
|  |  |  | Convinced that the GPC will work | "Administrative barrier given that, although not a straitjacket, they are not mandatory" (**PSSI**: 1). |  |
| C | Patient factors | Patient behavior | Knowledge about the  (preventive) role of health teams. | "The guideline is very clinical, it has no community focus" (**PSSI** 2). | "Patient education on warning signs and symptoms,  making clear to the patient the reasons why he/she might require re-consultation to avoid complications." |
|  |  |  |  | In addition, despite the constant patient education, the patient does not fully adhere to the recommendations, "Yes, the truth is that sometimes they do not understand, but we try to talk with a teaching that they understand from us and from the doctors aswell" (**FGP**: 8). | "Health Education, which is a different approach to the traditional approach that has been given, let's say, to what should be done in prevention, in education, which is information, education and communication" (**PSSI** 5). "We are taking care of him, we always try to give him this education" (**FGP**: 6), "When they leave the institution, we give them all that again, we give them their formula, we explain to them how they are going to be handled at  home, what the warning signs are" (**FGP**: 6). |
|  |  | Patient needs | More attention to the work of patient communities | "In theory the guide responds, but the 2010 Guide, may present a barrier, such as not being in the framework of the RIAS, it does not say exactly what the community should do. It does not have elements for the communities in practice", (**PSSI**: 4).  "There is a very important factor that has been in a negative way so that suddenly mothers or families who have had dengue have been the media, people who have lost credibility in medicine and then turn to a YouTube video and are not aware of those media." (**FGP**:7). | "To generate confidence in the patient so that he/she consults, for this reason it is necessary to reactivate the community managers, so that they can reappear and be the bridge between the health service and the community". (**PSSI** 4)  "From the nursing side, we educate the patient, so that they know how serious it is because some of them may get well, while others may get complicated and even die" (**FGP**:6), "there are others who go home and we continue with the education that exists in the families if they have the symptoms to warn them because they may have a focus there and they do not know and on the part of health to tell them to wash the sinks well that if they have a lot of worms or larvae to go and visit them... well, all  those little things" (**FGP**:6). |
| D | Professional interactions | Coordination and collaboration among health professionals | Reference and counter- reference | "The sustainability of the system takes precedence over patient care, given that the system does not work if the patient does not contribute, there are many difficulties with patient referrals in very distant municipalities. In these contexts, talking about referral and  counter-referral is another reality" (**PSSI**: 4). |  |
|  |  | Teamwork among professionals | Common/shared interest with stakeholders involved. | “An interprofessional training approach that I find interesting that is being supported and led by the Ministry of Health with the support of PAHO and from the different institutions of human resources in health, because unfortunately human resources have not been trained for teamwork, but have been trained so that each one does his part; we must then  identify those boundaries that exist in the work of health teams". (**PSSI**: 3). |  |
|  |  |  |  | "There is a lack of space in the training curricula, not only in the fields of public health, but also in other fields of health knowledge, in the field of nursing, in the field of microbiology before bacteriology, and of course in medicine, I would say” (**PSSI**. 3) |  |
|  |  |  |  | "In any case, the institutions, whether due to political, economic or social problems, be it the radio, the municipality, Chinita Clinic, Panamerican Clinic, work alone, in isolation; in other words, there is a lack of a path of action that is truly complied with where there is efficiency in the health sector". (**FGP**:8). |  |
|  |  | The influence of beliefs, ideas and communication among health professionals. | Little or no feedback on the actions of the health teams. | "Bacteriologists, they are in a laboratory and in practice they do not disseminate knowledge about diagnostic laboratory tests, nor do they provide feedback to healthcare personnel." |  |
|  |  |  | Inadequate transfer and exchange of information | "Tests for dengue diagnosis are scarce; bacteriologists have no contact with the clinical history, and their role does not go beyond taking a blood sample and processing it" (**PSSI**: 5). |  |
|  |  |  |  | "If, for example, you see a sign of alarm, you tell the physician what is happening with the patient, or the internist who is also in charge of the patient" (**FGP**:3), "There are some physicians who do not accept that you tell them that they send you the dengue test very early, and they do not accept that you make that recommendation" **FGP**:7). |  |
|  |  |  | Permanent rotation of health teams. | "Territorial entities modify human resources according to their interests and the change of government means that this change of personnel does not allow for continuity and local capacity building" (**PSSI** 1). | "It is articulated with the mayor's office for health interventions, brigades and extramural processes for patient education and to promote disease prevention". (**FGP**:7). |
| E | Incentives and resources | Availability of necessary resources | Responsibility of the central level in the implementation process | "The investment of resources in public health has historically been very limited compared to what has been invested in the curative approach to the disease; I highlight the great effort made by the National Institute of Health to strengthen public health surveillance and also to serve as a national reference laboratory, but I think it has remained very centralized" (**PSSI**: 2). |  |
|  |  |  |  | "The entities responsible for ensuring that there are appropriate conditions and capacities to provide a timely response to the management of dengue, if they themselves are not making the necessary efforts, it is very difficult for the personnel to take ownership, for the institutions themselves to take ownership, especially when they have a more curative and business approach”. (**PSSI**: 3). |  |
|  |  |  |  | "Last year the health secretary gave us training on dengue fever, but not for years and this year we have not had training on dengue fever" (**FGP**:8) "Since I have been working here there have been about two trainings on dengue fever, but more or less for about two years" (**FGP**:7) "in the past, when there was no pandemic, we were trained" (**FGP**:8). No one mentions an induction process or any other type of stimuli. |  |
|  |  | Positive and negative financial incentives | The dynamics of resources and incentives in the framework of the adaptation and adoption  of guides | "The country does not have the economic or personnel resources to carry out a process of adapting guides." (**PSSI**: 2). |  |
|  |  | Refresher training systems | More integrated work in basic medical training | "Health education projects are limited to informative talks. The CIP is focused on health education in the different environments. And the resources are there, but they are not used adequately due to lack  of execution". (**PSSI**: 1, 4). |  |
|  |  | Presence of practical tools for health professionals | No standardized tool is yet available | "How to incentivize doing things right, but I don't know how much I've really studied about that negative impact of the problem there is with the guidelines." (**PSSI** 5)  "Currently there is no stimulus, prior to the health emergency there was a training stimulus since academic talks were held twice a week which due to the pandemic and social distancing have not been held again." (**FGP**:8). |  |
| F | Capacity for change in organizations | Mandate and authority | The health system has a responsibility | "Structural difficulties that exist in the health system, so the guide in itself should be a facilitator as such, but it is simply where the barriers and facilitators are found, so the barriers are at all levels, they are from the community, from the families themselves who have to learn to realize when to run to the health institution because there is a sign of alarm, right, so that the health institution has the capacity to identify them, not to send people back as if it were any fever if there is an alarm sign and of course to provide timely care, to avoid decompensation and complications in cases with alarm signs, so, in this whole process, there are barriers that must be identified in this implementation research! (**PSSI**: 2). |  |
|  |  | Qualified leadership | Demanding attention for health service issues | "We are contributing to the spread of the disease and even generating cases of in-  hospital dengue" (**PSSI**:. 3). |  |
|  |  | Supporting force | Network of laboratories adapted to change | "The capacities in the network of laboratories and the healthcare providers themselves with clinical laboratories for the confirmation of cases, I believe, are still very limited including  serotype surveillance" (**PSSI**: 3). |  |
|  |  | Regulations, rules, policies in  the territories | Advice for the territories | "A lot of fragmentation within the institutions and a lack of capacity for integrality in the work at the territorial level" (**PSSI**: 3). |  |
|  |  | Help needed with change in organizations | Appropriation of the guidelines generated by the CPG. | "The guide responds to the prevention aspects: The key points if contained, but they are not appropriated by the health professional, much less by the patient and family members." (**PSSI**: 5) |  |
|  |  |  |  | "There is no follow-up supervision of guides, there is no one in charge of this, but we do self-monitoring." (**FGP**: 2) |  |
|  |  | Monitoring and feedback | Inconsistency in the orientation of the health teams, in compliance with the CPGs. | "The conviction in the guidelines really, I think what is missing is that, that we really follow the guidelines, because we are convinced what it is and what should be done and not like...well...I do it if I want to..., says the lab" (**PSSI**: 5). |  |
|  |  |  |  | "There is no institutional policy to follow up on the process of adherence to dengue guidelines, nor is it an institutional priority within its organizational processes" (**FGP**:10). |  |
|  |  |  |  | "Let's not lie to ourselves, adherence to the guidelines is more of a form of audit view than of training. I think it is valid, I do not deny it; but it is everywhere, I am not questioning the institutions, it is the parameter that is used to verify adherence to the guidelines with a parameter of audit rather than of sensitivity, the system itself and the guidelines, that is the question". (**FGP**:9) |  |
| G | Social, political and legal  aspects | Regulation and legislation | Ignorance of regulations, rights and  obligations | "The legal process that supports the 2010 guide limits the application of updated guidelines or guidelines more in line with the current reality of the country. For this reason, we are still anchored to the 2010 guidelines” (**PSSI**: 1) |  |
|  |  | Financing policy | Insufficient support to decision makers | "The hospital infrastructure itself for patient care, there are many, many deficiencies." (**PSSI**: 3) |  |
|  |  |  |  | "A major limitation in terms of the capacity of the Ministry and also of the Institute to accompany, to advise, to monitor and control that a good job is being done at the level of the territories and in this time of pandemic, well, I would say that a good part of this work was practically abandoned" (**PSSI**: 3). |  |
|  |  |  | Public funding is needed for academic and public health research. |  | "Research institutions or academic institutions have a very important job to do in articulated work with the institutions, with the health authorities of the territorial entities and even among themselves; many times there are also competencies among the same academic and research institutions or in channeling resources that also fragment the possibility of having an accompaniment that produces a better impact on the response that should be given to problems such as dengue" (**PSSI** 3). |
|  |  |  | Financing policy for municipalities | "There are no resources to reach communities, Communities must be strengthened to improve local capacity" (**PSSI** 1). |  |
|  |  | Political stability | Influence of political decision making | "Clinical guidelines are made looking towards the hills of Bogota, but with their backs to the country". (**PSSI** 1) |  |
|  |  | Adaptation process vs. adoption of GPC | Implement new models |  | "The country is going to adopt the latest guideline issued by PAHO for the Americas because of the good quality of its sources and it is the only one in the world for arboviruses. It is something very positive because we include Chikungunya and Zika, we have evolution in relation to pediatric patient management, we see it as a great opportunity for the country" (**PSSI** 1). |

**GACIPD**: Guía de Atención Integral del Dengue (Comprehensive Care Guide for Dengue)

**PSSI**: Participant in the semi-structured interview

**FGP**: Focus Group Participant.

**CPG**: Clinical Practice Guideline

**Supplementary Material S4**

**Table B: Results of focus groups**

| Number | Domain | Determinant | Subject | Barriers | Facilitators |
| --- | --- | --- | --- | --- | --- |
| A | Guideline Factors: GACIPD | How GACIPD  was developed | To be in line with current developments in GPC design | "The guide is not very clear when a patient has renal failure, when a patient is diabetic, when a patient is hypertensive, when a patient has thousands of infarctions and has dengue, and the guide is not clear on that, or when it comes with co-infections, leptospirosis, malaria" (**FGP**:7). | "The guidance is very clear in saying what to do with a patient who only has dengue," **FGP**:7). |
|  |  | To be able to work with GACIPD | To be in line with the current form of consultation in GPC. | Difficulty in the diagnostic approach due to the different differential diagnoses existing in the Urabá region of etiologies of febrile syndrome. | "It seems to me that the guide is very important to analyze not only the clinical context, but also the social context, psychology", (**FGP**:2). |
|  |  |  | Experimenting/practicing with GPCs | "I have no knowledge about the guide, but I know something about dengue" (**FGP**:1), "we don't read the guide like that" (**FGP**:2) "regarding the guide is that I have realized that nobody knows it, but they know what they talk about here" (**FGP**:10). | "I think it is a clear, concise, precise guide." (**FGP**:1). |
|  |  | Behavior required for the use of the GACIPD | Incorporate in local guidelines | "Basically there was not a socialization as such that guides the protocol" (**FGP**:10), "In the year before last the health secretariat gave us a training on dengue, but not for years back in the year we have not had training on dengue" (**FGP**:8), "a guideline as such does not exist, I was told when you have a suspected patient what you have to do is based on what you already have as knowledge of the pathology"  (**FGP**:10). | The medical knowledge accumulated by practicing in a dengue endemic area (FGP: 8). |
| B | Individual health professional factors | Knowledge and skills of health teams. | Professionals are not familiar with the subject | "Generally, now there are many new doctors, so doctors rotate a lot, today we have one, tomorrow we have another...trained, I say, by certain pathologies to which they adhere, so that is very difficult" (**FGP**: 8). |  |
|  |  | Knowledge, beliefs and attitudes of health teams. | Attention to individual needs and competencies | "A guideline as such does not exist, I was told that when you have a suspicious patient, what you have to do is based on what you already know about the pathology" (**FGP**:10). | Carrying out training in health posts far from the institution (FGP: 8). |
|  |  |  |  | "So I think that if we are going to look at this guide, it lacks, it lacks patients with comorbidities and if it says a little bit over there it is very brief and we know and understand that they are more complicated, which are precisely those who have comorbidities." (**FGP**:4) |  |
|  |  | Professional behavior of health teams | Self-confidence in one's own ability |  |  |
|  |  |  | Convinced that the GPC will work |  |  |
| C | Patient factors | Patient behavior | Knowledge about the  (preventive) role of health teams. | In addition, despite the constant patient education, the patient does not fully adhere to the recommendations, "Yes, the truth is that sometimes they do not understand, but we try to talk with a teaching that they understand from us and from the doctors as well" (**FGP**: 8). | "Patient education on warning signs and symptoms,  making clear to the patient the reasons why he/she might require re-consultation to avoid complications." |
|  |  |  |  |  | "We are taking care of him, we always try to give him this education" (**FGP**: 6), "When they leave the institution, we give them all that again, we give them their formula, we explain to them how they are going to be handled at  home, what the warning signs are" (**FGP**: 6). |
|  |  | Patient needs | More attention to the work of patient communities | "There is a very important factor that has been in a negative way so that suddenly mothers or families who have had dengue have been the media, people who have lost credibility in medicine and then turn to a YouTube video and are not aware of those media." (**FGP**:7). | "From the nursing side, we educate the patient, so that they know how serious it is because some of them may get well, while others may get complicated and even die" (**FGP**:6), "there are others who go home and we continue with the education that exists in the families if they have the symptoms to warn them because they may have a focus there and they do not know and on the part of health to tell them to wash the sinks well that if they have a lot of worms or larvae to go and visit them... well, all  those little things" (**FGP**:6). |
| D | Professional interactions | Coordination and collaboration among health professionals | Reference and counter- reference |  | Teamwork in the institution and good medical coordination, assertive communication regardless of one's professional level. (FGP: 8). |
|  |  | Teamwork among professionals | Common/shared interest with stakeholders involved. | "In any case, the institutions, whether due to political, economic or social problems, be it the radio, the municipality, Chinita Clinic, Panamerican Clinic, work alone, in isolation; in other words, there is a lack of a path of action that is truly complied with where there is efficiency in the health sector". (**FGP**:8). |  |
|  |  | The influence of beliefs, ideas and communication among health professionals. | Little or no feedback on the actions of the health teams. | "Bacteriologists, they are in a laboratory and in practice they do not disseminate knowledge about diagnostic laboratory tests, nor do they provide feedback to healthcare personnel." | The interdisciplinary work both in the processes of filling out the file and notification and in the processes of carrying out laboratories test and results (FGP:3) |
|  |  |  | Inadequate transfer and exchange of information | "If, for example, you see a sign of alarm, you tell the physician what is happening with the patient, or the internist who is also in charge of the patient" (**FGP**:3), "There are some physicians who do not accept that you tell them that they send you the dengue test very early, and they do not accept that you make that recommendation" **FGP**:7). |  |
|  |  |  | Permanent rotation of health teams. |  | "It is articulated with the mayor's office for health interventions, brigades and extramural processes for patient education and to promote disease prevention". (**FGP**:7). |
| E | Incentives and resources | Availability of necessary resources | Responsibility of the central level in the implementation process | "Last year the health secretary gave us training on dengue fever, but not for years and this year we have not had training on dengue fever" (**FGP**:8) "Since I have been working here there have been about two trainings on dengue fever, but more or less for about two years" (**FGP**:7) "in the past, when there was no pandemic, we were trained" (**FGP**:8). No one mentions an induction process or any other type of stimuli. | There is an institutional laboratory, with rapid performance of laboratory tests, on an opportune basis, especially in patients with probable dengue. (FGP:3) |
|  |  | Positive and negative financial incentives | The dynamics of resources and incentives in the framework of the adaptation and adoption  of guides | Barriers in the administrative part to access the NS1 antigen test. The lack of a rapid dengue test in rural areas where the patient, due to geographical and economic difficulties, has a delay in diagnosis and treatment. (FGP:8). |  |
|  |  | Refresher training systems | More integrated work in basic medical training |  |  |
|  |  | Presence of practical tools for health professionals | No standardized tool is yet available | "Currently there is no stimulus, prior to the health emergency there was a training stimulus since academic talks were held twice a week which due to the pandemic and social distancing have not been held again." (**FGP**:8). |  |
| F | Capacity for change in organizations | Mandate and authority | The health system has a responsibility | Institutional policies based on production and not on the quality of health personnel (FGP:10). | Institutional support from the prevention and promotion, surveillance, nursing, and quality group, with early alerts and monitoring of clinical and case records. (FGP:10). |
|  |  | Qualified leadership | Demanding attention for health service issues | Lack of institutional policies and monitoring of the guideline adherence process. (FGP:8). |  |
|  |  | Supporting force | Network of laboratories adapted to change |  |  |
|  |  | Regulations, rules, policies in  the territories | Advice for the territories | Lack of a solid institutional guideline that is not affected by the frequent changes in administration of the institution. (FGP:10). |  |
|  |  | Help needed with change in organizations | Appropriation of the guidelines generated by the CPG. | "There is no follow-up supervision of guides, there is no one in charge of this, but we do self-monitoring." (**FGP**: 2) | The availability of the guide on the institutional platform. (FGP:10). |
|  |  | Monitoring and feedback | Inconsistency in the orientation of the health teams, in compliance with the CPGs. |  |  |
|  |  |  |  | "There is no institutional policy to follow up on the process of adherence to dengue guidelines, nor is it an institutional priority within its organizational processes" (**FGP**:10). | The standardization of processes and the unification of knowledge for its application with patient care. (FGP: 2) |
|  |  |  |  | "Let's not lie to ourselves, adherence to the guidelines is more of a form of audit view than of training. I think it is valid, I do not deny it; but it is everywhere, I am not questioning the institutions, it is the parameter that is used to verify adherence to the guidelines with a parameter of audit rather than of sensitivity, the system itself and the guidelines, that is the question". (**FGP**:9) |  |
| G | Social, political and legal  aspects | Regulation and legislation | Ignorance of regulations, rights and  obligations |  |  |
|  |  | Financing policy | Insufficient support to decision makers | Increase in the burden of care for the uninsured population (migrants, indigenous people) (FGP:9) |  |
|  |  |  | Public funding is needed for academic and public health research. | Non-availability in the Pediatric Intensive Care Unit area. (FGP:9) |  |
|  |  |  | Financing policy for municipalities |  |  |
|  |  | Political stability | Influence of political decision making | Lack of an updated diagnostic algorithm in emergency services. (FGP:9) |  |
|  |  | Adaptation process vs. adoption of GPC | Implement new models | Limitations to timely access to health due to geographical and economic barriers (FGP:9) |  |

**GACIPD**: Guía de Atención Integral del Dengue (Comprehensive Care Guide for Dengue)

**FGP**: Focus Group Participant.

**CPG**: Clinical Practice Guideline

**Table C: Results of the semi-structured interviews**

| Number | Domain | Determinant | Subject | Barriers | Facilitators |
| --- | --- | --- | --- | --- | --- |
| A | Guideline Factors: GACIPD | How GACIPD  was developed | To be in line with current developments in GPC design | "It is unknown that a guide is elaborated by experts and validated from evidence-based medicine, a lack of knowledge that comes from the undergraduate training programs" (**PSSI**:7). |  |
|  |  | To be able to work with GACIPD | To be in line with the current form of consultation in GPC. | It is also mentioned that the guide is outdated "The Dengue Guide is outdated. It is a guide that is not in line with current regulations, with the regulatory and administrative process"  (**PSSI**.1,3,4,5,6). |  |
|  |  | Behavior required for the use of the GACIPD | Incorporate in local guidelines | "The current guide is 11 years old, very academic, but with big gaps and there have been regulatory and (PIC, institutional environment, work etc., integrated management, RIAS, Rapid tests, resolution 3280) administrative changes that are not reflected in these guides. It is a guide that is not in line with current regulations, with the regulatory and administrative process. There is a great disadvantage in not updating the guidelines on a permanent basis". (**PSSI**: 1) |  |
| B | Individual health professional factors | Knowledge and skills of health teams. | Professionals are not familiar with the subject | "Well, how important is it from the laboratory's point of view that they do the test that is much more sensitive and that we do not have it in the institution, I understand that it is a limitation for us as physicians" (**PSSI**: 3). | they can be resolved through ICT strategies: videos, virtual spaces, podcasts, listening to what people want to be explained, through images and listening" (**PSSI**: 6). ICTs: Information and communication technologies. |
|  |  | Knowledge, beliefs and attitudes of health teams. | Attention to individual needs and competencies | “From undergraduate training there is not much emphasis for knowledge of the guide" (**PSSI**: 6).  "There are students already graduated from the undergraduate program who come with many gaps in their knowledge about the dengue guide"; (**PSSI**:5).  "In medical schools they are not made known, they are only known in practice when faced with a patient." (**PSSI:** 4) |  |
|  |  | Professional behavior of health teams | Self-confidence in one's own ability | "It is necessary to demystify that guidelines take away autonomy, in other fields such as nursing; I have seen it in medicine: ah, but then where is my knowledge, my autonomy to treat patients, as I believe? if we manage to break that scheme in the feeling, we can overcome the saying, is that the doctor, nurse,  nurse or microbiologist is autonomous." (**PSSI:**1). |  |
|  |  |  | Convinced that the GPC will work | "Administrative barrier given that, although not a straitjacket, they are not mandatory" (**PSSI**: 1). |  |
| C | Patient factors | Patient behavior | Knowledge about the  (preventive) role of health teams. | "The guideline is very clinical, it has no community focus" (**PSSI** 2). | "Patient education on warning signs and symptoms, making clear to the patient the reasons why he/she might require re-consultation to avoid complications." |
|  |  |  |  |  | "Health Education, which is a different approach to the traditional approach that has been given, let's say, to what should be done in prevention, in education, which is information, education and communication" (**PSSI** 5 |
|  |  | Patient needs | More attention to the work of patient communities | "In theory the guide responds, but the 2010 Guide, may present a barrier, such as not being in the framework of the RIAS, it does not say exactly what the community should do. It does not have elements for the communities in practice", (**PSSI**: 4). | "To generate confidence in the patient so that he/she consults, for this reason it is necessary to reactivate the community managers, so that they can reappear and be the bridge between the health service and the community". (**PSSI** 4) |
| D | Professional interactions | Coordination and collaboration among health professionals | Reference and counter- reference | "The sustainability of the system takes precedence over patient care, given that the system does not work if the patient does not contribute, there are many difficulties with patient referrals in very distant municipalities. In these contexts, talking about referral and  counter-referral is another reality" (**PSSI**: 4). |  |
|  |  | Teamwork among professionals | Common/shared interest with stakeholders involved. | “An interprofessional training approach that I find interesting that is being supported and led by the Ministry of Health with the support of PAHO and from the different institutions of human resources in health, because unfortunately human resources have not been trained for teamwork, but have been trained so that each one does his part; we must then  identify those boundaries that exist in the work of health teams". (**PSSI**: 3). |  |
|  |  |  |  | "There is a lack of space in the training curricula, not only in the fields of public health, but also in other fields of health knowledge, in the field of nursing, in the field of microbiology before bacteriology, and of course in medicine, I would say” (**PSSI**. 3) |  |
|  |  | The influence of beliefs, ideas and communication among health professionals. | Little or no feedback on the actions of the health teams. | "Bacteriologists, they are in a laboratory and in practice they do not disseminate knowledge about diagnostic laboratory tests, nor do they provide feedback to healthcare personnel." |  |
|  |  |  | Inadequate transfer and exchange of information | "Tests for dengue diagnosis are scarce; bacteriologists have no contact with the clinical history, and their role does not go beyond taking a blood sample and processing it" (**PSSI**: 5). |  |
|  |  |  | Permanent rotation of health teams. | "Territorial entities modify human resources according to their interests and the change of government means that this change of personnel does not allow for continuity and local capacity building" (**PSSI** 1). |  |
| E | Incentives and resources | Availability of necessary resources | Responsibility of the central level in the implementation process | "The investment of resources in public health has historically been very limited compared to what has been invested in the curative approach to the disease; I highlight the great effort made by the National Institute of Health to strengthen public health surveillance and also to serve as a national reference laboratory, but I think it has remained very centralized" (**PSSI**: 2). |  |
|  |  |  |  | "The entities responsible for ensuring that there are appropriate conditions and capacities to provide a timely response to the management of dengue, if they themselves are not making the necessary efforts, it is very difficult for the personnel to take ownership, for the institutions themselves to take ownership, especially when they have a more curative and business approach”. (**PSSI**: 3). |  |
|  |  | Positive and negative financial incentives | The dynamics of resources and incentives in the framework of the adaptation and adoption  of guides | "The country does not have the economic or personnel resources to carry out a process of adapting guides." (**PSSI**: 2). |  |
|  |  | Refresher training systems | More integrated work in basic medical training | "Health education projects are limited to informative talks. The CIP is focused on health education in the different environments. And the resources are there, but they are not used adequately due to lack  of execution". (**PSSI**: 1, 4). |  |
|  |  | Presence of practical tools for health professionals | No standardized tool is yet available | "How to incentivize doing things right, but I don't know how much I've really studied about that negative impact of the problem there is with the guidelines." (**PSSI** 5) |  |
| F | Capacity for change in organizations | Mandate and authority | The health system has a responsibility | "Structural difficulties that exist in the health system, so the guide in itself should be a facilitator as such, but it is simply where the barriers and facilitators are found, so the barriers are at all levels, they are from the community, from the families themselves who have to learn to realize when to run to the health institution because there is a sign of alarm, right, so that the health institution has the capacity to identify them, not to send people back as if it were any fever if there is an alarm sign and of course to provide timely care, to avoid decompensation and complications in cases with alarm signs, so, in this whole process, there are barriers that must be identified in this implementation research! (**PSSI**: 2). |  |
|  |  | Qualified leadership | Demanding attention for health service issues | "We are contributing to the spread of the disease and even generating cases of in-  hospital dengue" (**PSSI**:. 3). |  |
|  |  | Supporting force | Network of laboratories adapted to change | "The capacities in the network of laboratories and the healthcare providers themselves with clinical laboratories for the confirmation of cases, I believe, are still very limited including serotype surveillance" (**PSSI**: 3). |  |
|  |  | Regulations, rules, policies in  the territories | Advice for the territories | "A lot of fragmentation within the institutions and a lack of capacity for integrality in the work at the territorial level" (**PSSI**: 3). |  |
|  |  | Help needed with change in organizations | Appropriation of the guidelines generated by the CPG. | "The guide responds to the prevention aspects: The key points if contained, but they are not appropriated by the health professional, much less by the patient and family members." (**PSSI**: 5) |  |
|  |  |  |  |  |  |
|  |  | Monitoring and feedback | Inconsistency in the orientation of the health teams, in compliance with the CPGs. | "The conviction in the guidelines really, I think what is missing is that, that we really follow the guidelines, because we are convinced what it is and what should be done and not like...well...I do it if I want to..., says the lab" (**PSSI**: 5). |  |
| G | Social, political and legal  aspects | Regulation and legislation | Ignorance of regulations, rights and  obligations | "The legal process that supports the 2010 guide limits the application of updated guidelines or guidelines more in line with the current reality of the country. For this reason, we are still anchored to the 2010 guidelines” (**PSSI**: 1) |  |
|  |  | Financing policy | Insufficient support to decision makers | "The hospital infrastructure itself for patient care, there are many, many deficiencies." (**PSSI**: 3) |  |
|  |  |  |  | "A major limitation in terms of the capacity of the Ministry and also of the Institute to accompany, to advise, to monitor and control that a good job is being done at the level of the territories and in this time of pandemic, well, I would say that a good part of this work was practically abandoned" (**PSSI**: 3). |  |
|  |  |  | Public funding is needed for academic and public health research. |  | "Research institutions or academic institutions have a very important job to do in articulated work with the institutions, with the health authorities of the territorial entities and even among themselves; many times there are also competencies among the same academic and research institutions or in channeling resources that also fragment the possibility of having an accompaniment that produces a better impact on the response that should be given to problems such as dengue" (**PSSI** 3). |
|  |  |  | Financing policy for municipalities | "There are no resources to reach communities, Communities must be strengthened to improve local capacity" (**PSSI** 1). |  |
|  |  | Political stability | Influence of political decision making | "Clinical guidelines are made looking towards the hills of Bogota, but with their backs to the country". (**PSSI** 1) |  |
|  |  | Adaptation process vs. adoption of GPC | Implement new models |  | "The country is going to adopt the latest guideline issued by PAHO for the Americas because of the good quality of its sources and it is the only one in the world for arboviruses. It is something very positive because we include Chikungunya and Zika, we have evolution in relation to pediatric patient management, we see it as a great opportunity for the country" (**PSSI** 1). |

**GACIPD**: Guía de Atención Integral del Dengue (Comprehensive Care Guide for Dengue)

**PSSI**: Participant in the semi-structured interview

**CPG**: Clinical Practice Guideline
