# Supplementary material for: Practice determinants for adherence to the Guide for the Comprehensive Clinical Care of Dengue Patients, Urabá (Colombia). A multifaceted approach to implementation research
Source: PLoS Negl Trop Dis. 2024 Aug 15;18(8):e0012361. doi: 10.1371/journal.pntd.0012361 (PMC11349210; doi:10.1371/journal.pntd.0012361)
Supplement: S5 Appendix — Distribution of the components of each of the 7 domains and score according to Likert scale. Domain A. Guide determinants. Domain B. Individual factors of health professionals. Domain C. Patient factors. Domain D. Professional Interactions. Domain E. Incentives and resources. Domain F. Capacity for organizational change. Domain G. Social, political, and legal factors. (DOCX) [file pntd.0012361.s007.docx]

**Supplementary Material S5**

Figures: Distribution of the components of each of the 7 domains and score according to Likert scale.

**Domain A: Guide determinants**

**
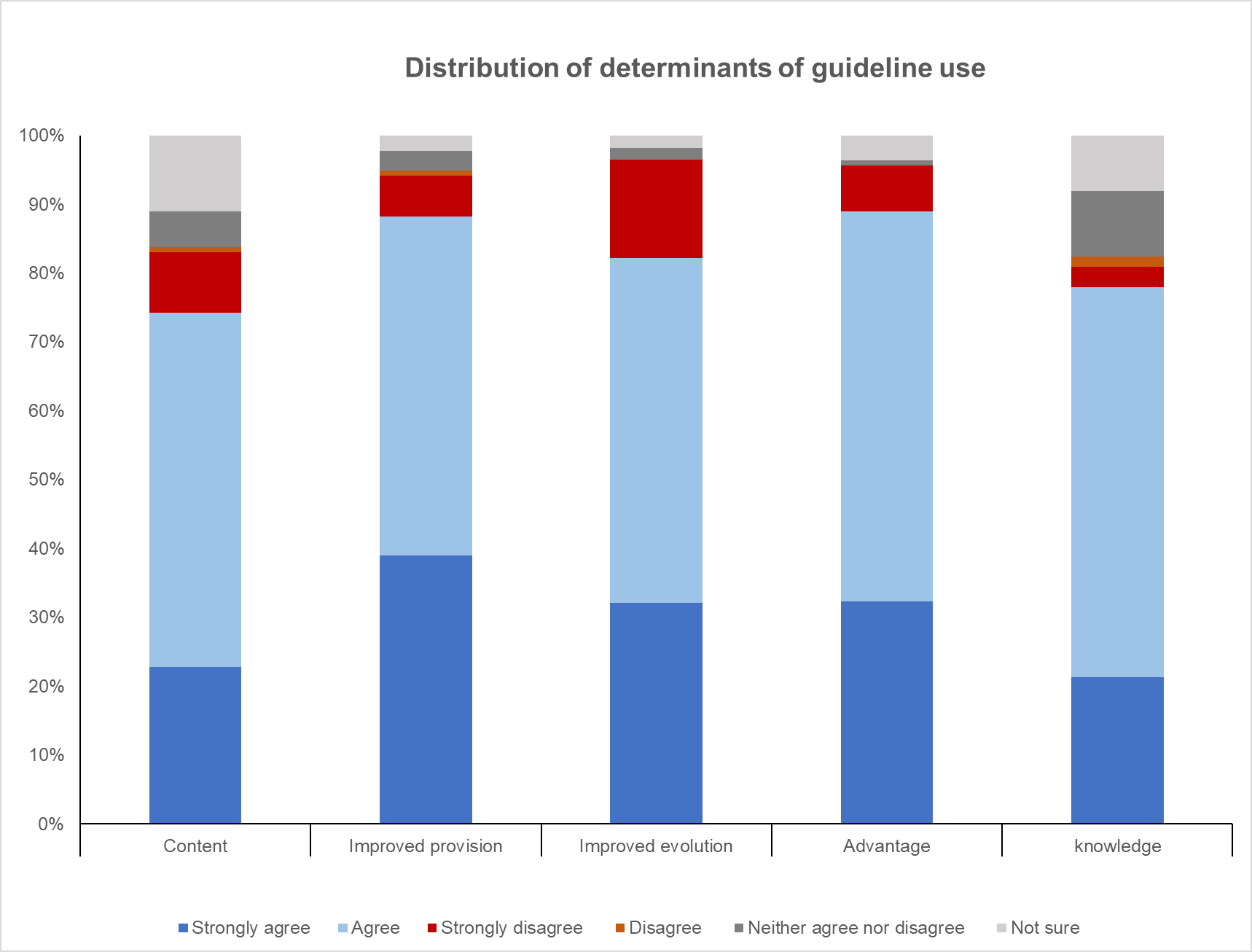
**

**Figure 1A: Distribution of determinants of guideline use (content, knowledge)**

**
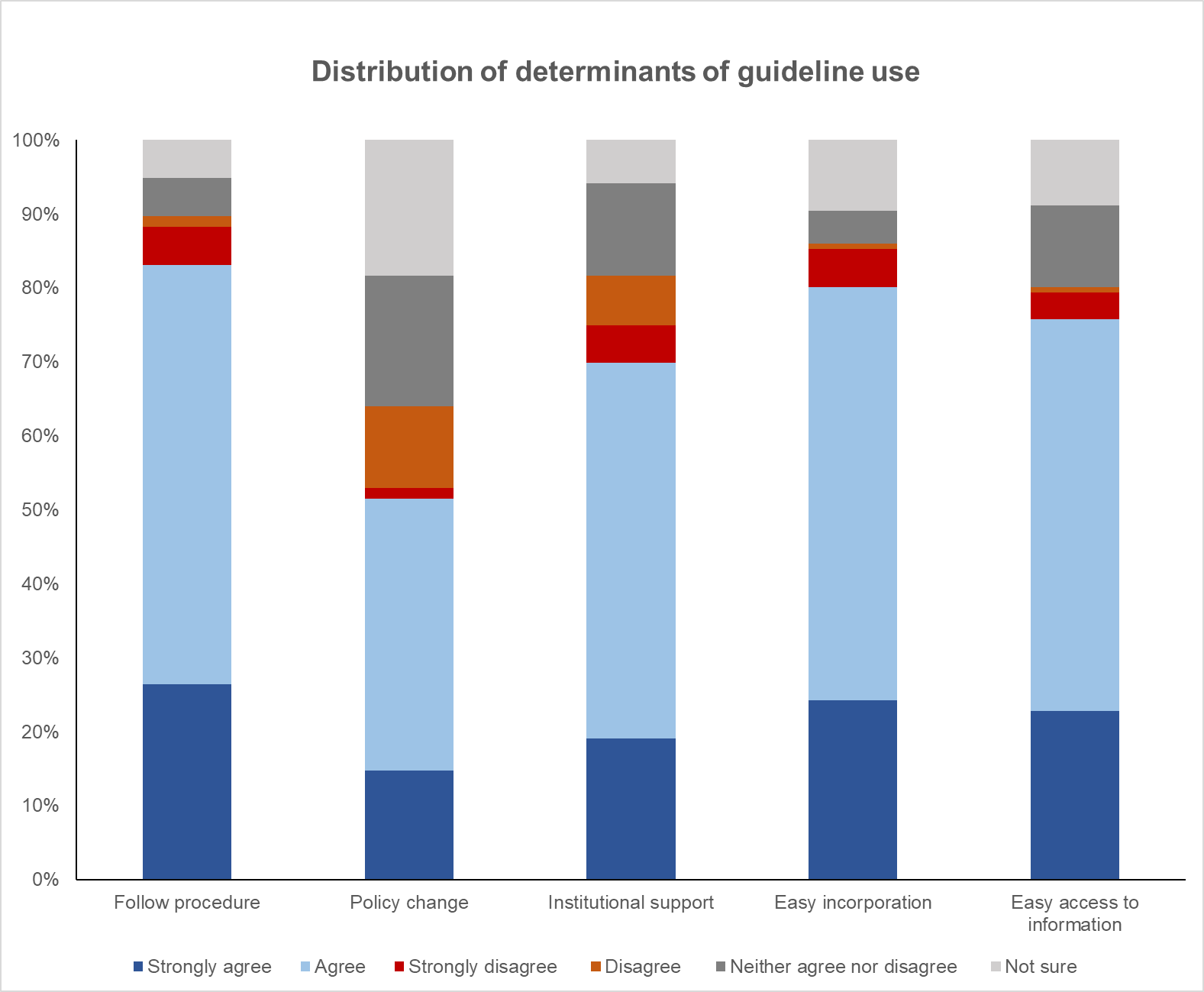
**

**Figure 1B: Distribution of determinants of guideline use (Follow procedure, Policy change)**

**
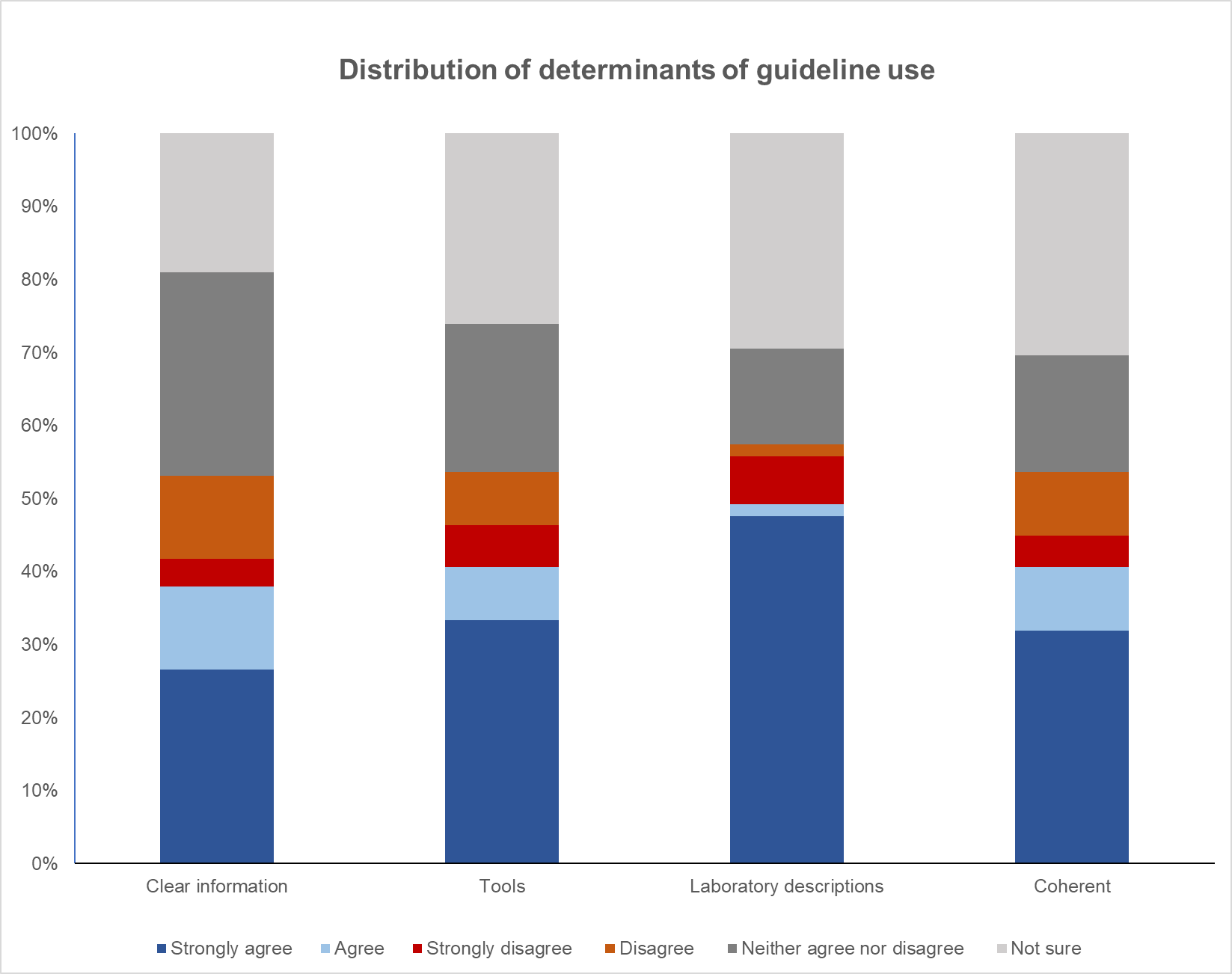
**

**Figure 1C: Distribution of determinants of guideline use (clear information, tools)**


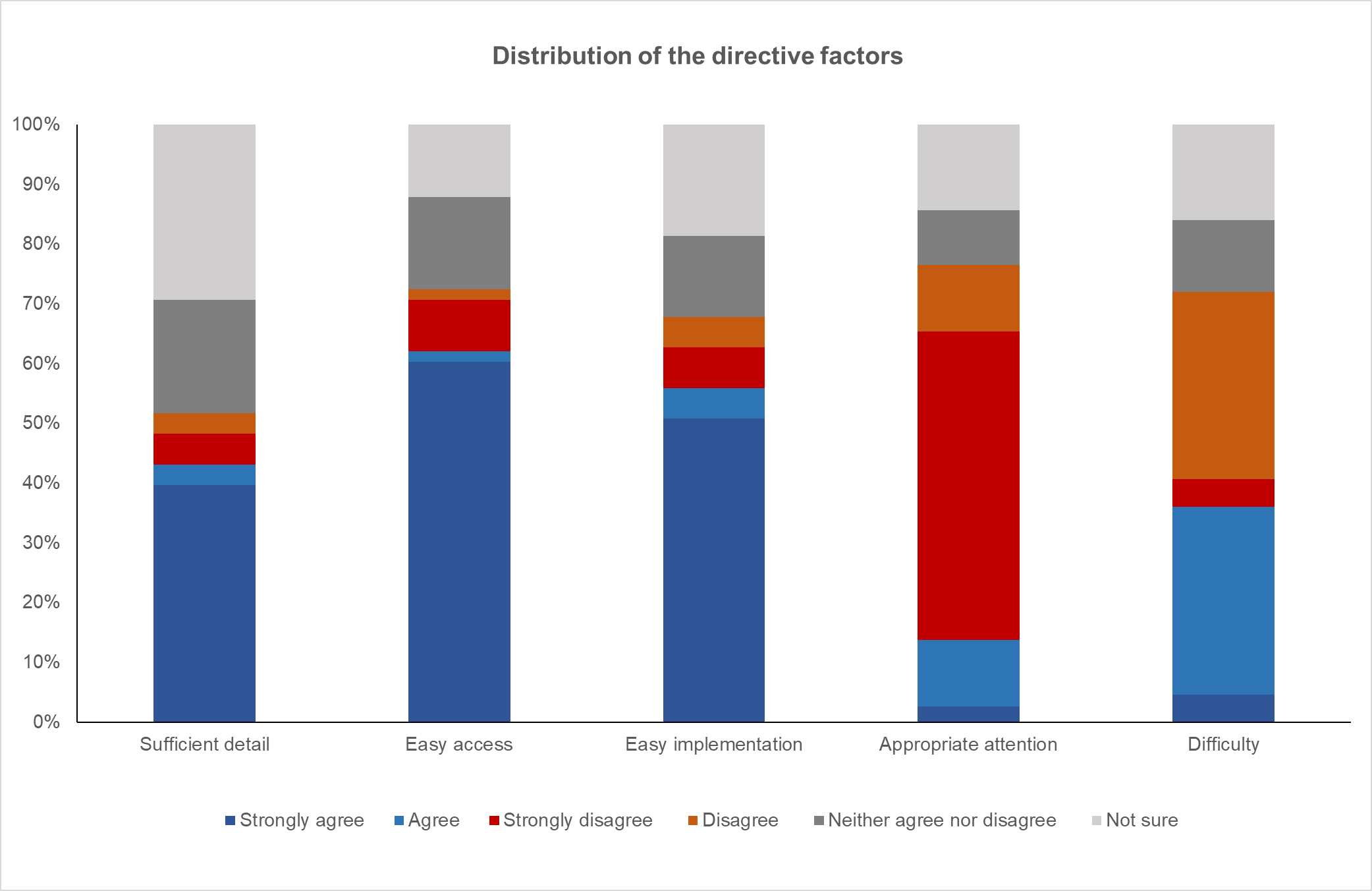


**Figure 2: Participants' perceptions about the guide and its use**


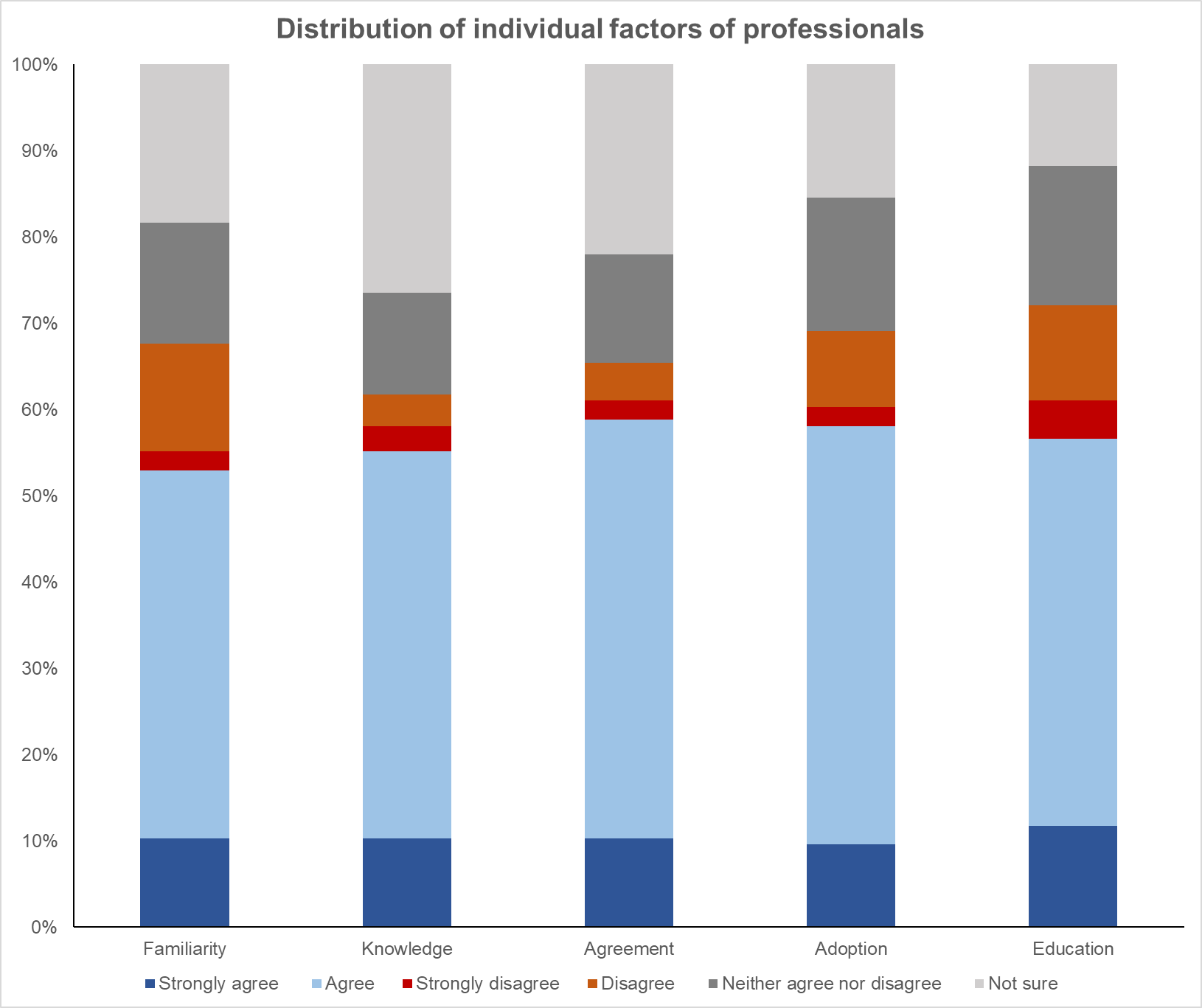
**Domain B: Individual factors of health professionals**

**Figure 3: Perception of individual factors of professionals**

**Domain C: Patient factors**


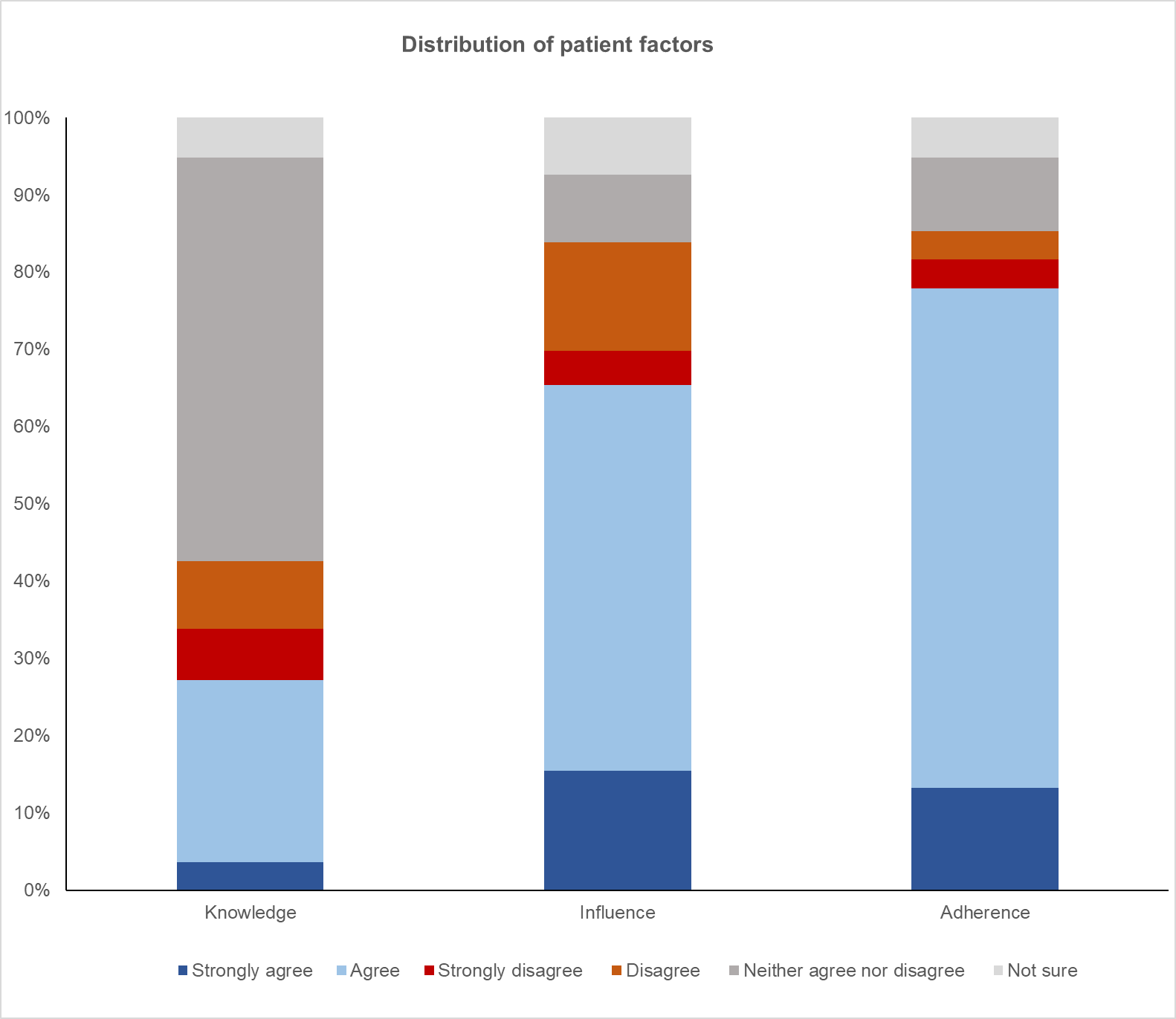


**Figure 5: Perception of Patient Factors**


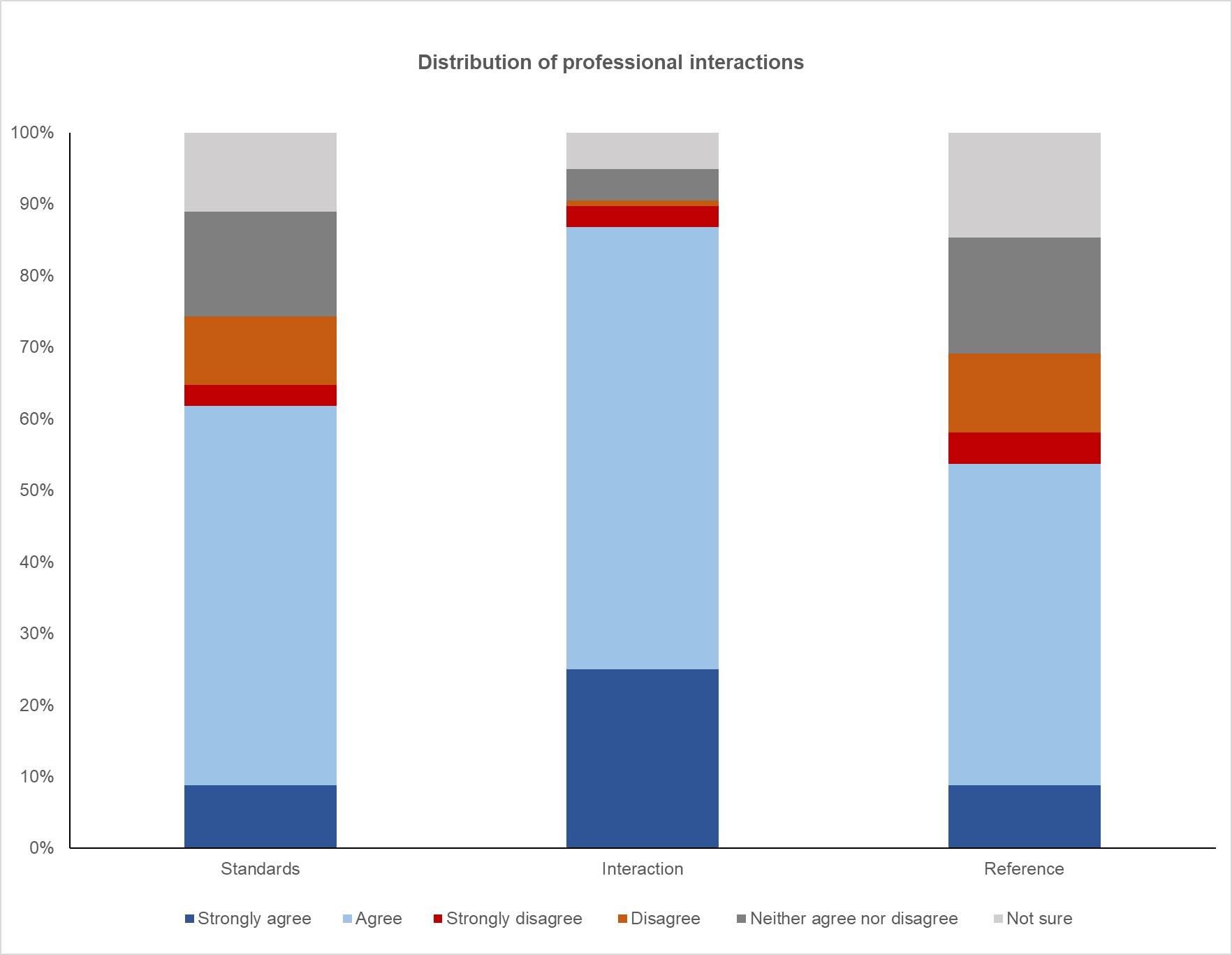
**Domain D: Professional Interactions**

**Figure 6: Perception of professional interactions**


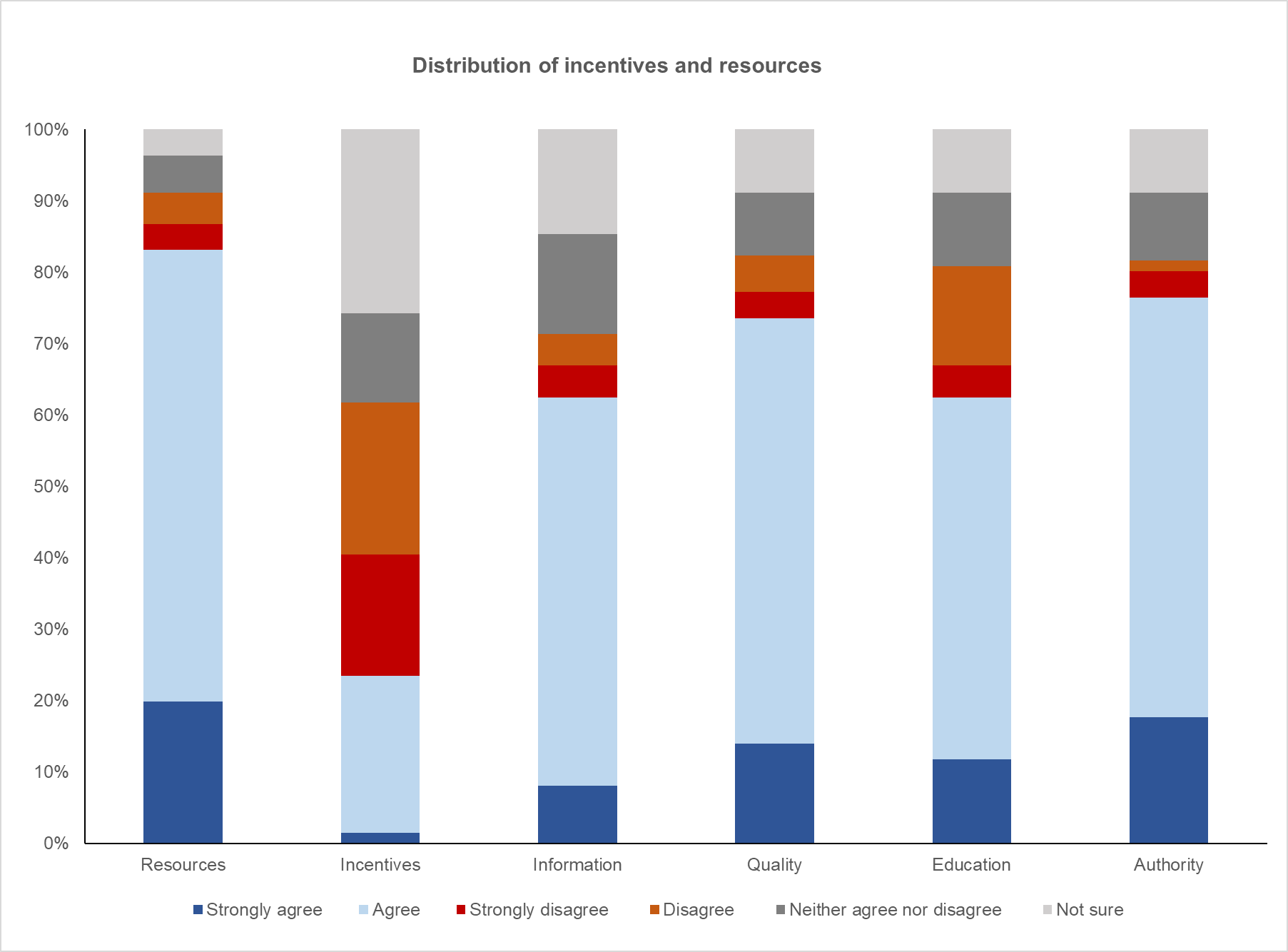
**Domain E: Incentives and resources**

**Figure 7: Perception of incentives and resources**


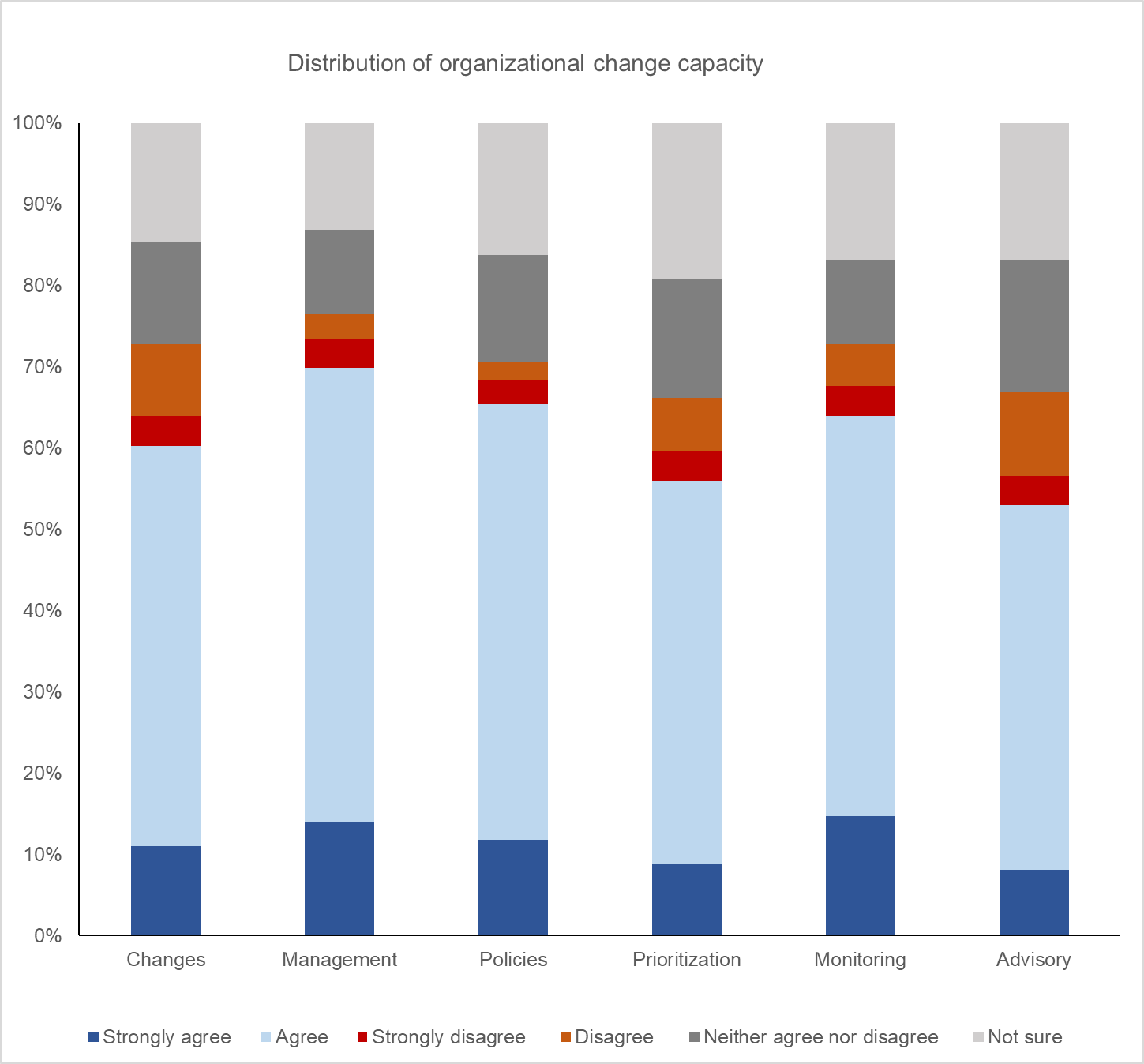
**Domain F: Capacity for organizational change**

**Figure 8: Perception of organizational change capacity**


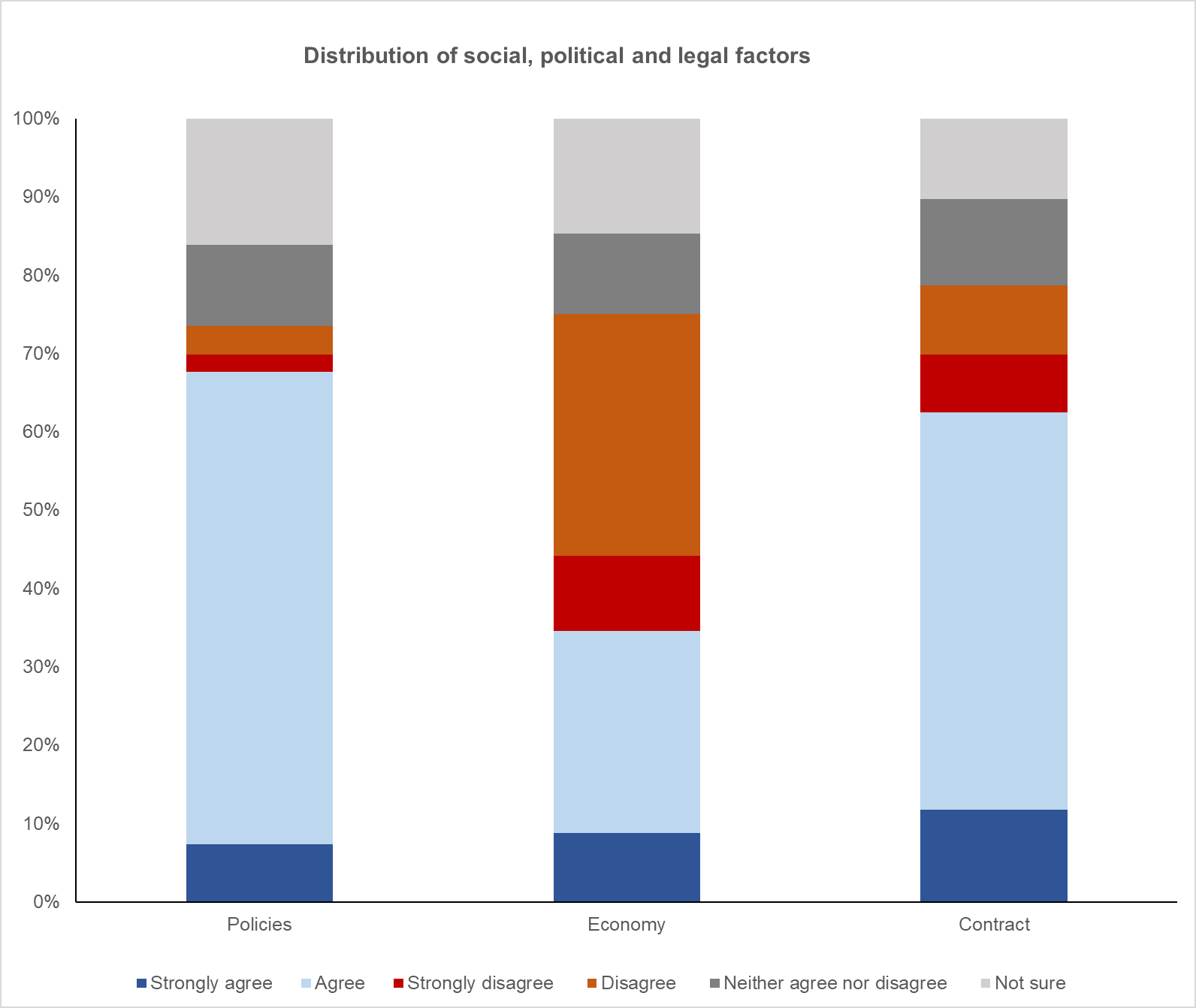
**Domain G:** **Social, political, and legal factors**

**Figure 9: Perception of social, political, and legal factors**
