# Supplementary material for: Practice determinants for adherence to the Guide for the Comprehensive Clinical Care of Dengue Patients, Urabá (Colombia). A multifaceted approach to implementation research
Source: PLoS Negl Trop Dis. 2024 Aug 15;18(8):e0012361. doi: 10.1371/journal.pntd.0012361 (PMC11349210; doi:10.1371/journal.pntd.0012361)
Supplement: S6 Appendix — Table A. Visualization of quantitative and qualitative results for determinants of GACIPD adherence. Urabá 2021. (DOCX) [file pntd.0012361.s008.docx]

**Supplementary Material S6**

**Table A: Visualization of quantitative and qualitative results for determinants of GACIPD adherence. Urabá 2021**

| **DOMAIN** | **CATEGORY** | **MEAN** | **SD** | **TESTIMONY** |
| --- | --- | --- | --- | --- |
|  | **RANGE SCORE** |  |  |  |
| D A | 18-42 LOW  43-66 MEDIUM 67-90 HIGH | 66,7 | 14,0 | "the guide is very clear in saying what to do with a patient who only has dengue, but the guide is not very clear when a patient has renal failure, when a patient is diabetic, when a patient is hypertensive, when a patient has thousands of infarctions and has dengue, and the guide is not clear on that, or when it comes with co-infections, leptospirosis, malaria" (**FGP**:7). |
|  |  |  |  | "I have no knowledge about the guide, but I know something about dengue" (**FGP**:1), "we don't read the guide like that" (**FGP**:2) "regarding the guide is that I have realized that nobody knows it, but they know what they talk about here" (**FGP**:10). |
| D B | 6-14 LOW 15-21 MEDIUM 22-30 HIGH | 18,3 | 5,8 | "In medical schools they are not made known, they are only known in practice when faced with a patient." (**PSSI**: 4) |
|  |  |  |  | "There are students already graduated from the undergraduate program who come with many gaps in their knowledge about the dengue guide"; (**PSSI**: 5). |
| D C | 5-11 LOW 12-18 MEDIUM 19-25 HIGH | 17,5 | 4,0 | "The guideline is very clinical; it has no community focus" (**PSSI**: 2). |
|  |  |  |  | "In theory the guide responds, but the 2010 Guide, may present a barrier, such as not being in the framework of the RIAS, it does not say exactly what the community should do. It does not have elements for the communities in practice", (**PSSI**: 4). |
| D D | 2-4 LOW 5-6 MEDIUM 7-10 HIGH | 6,0 | 1,7 | "There is a lack of space in the training curricula, not only in the fields of public health, but also in other fields of health knowledge, in the field of nursing, in the field of microbiology before bacteriology, and of course in medicine, I would say" (**PSSI**: 3). |
|  |  |  |  | "Tests for dengue diagnosis are scarce; bacteriologists have no contact with the clinical history, and their role does not go beyond taking a blood sample and processing it" (**PSSI**: 5). |
| D E | 7-16 LOW 17-25 MEDIUM 26-35 HIGH | 24,0 | 6,1 | "The country does not have the economic or personnel resources to carry out a process of adapting guides." (**PSSI**: 2) |
|  |  |  |  | "Health education projects are limited to informative talks. The CIP is focused on health education in the different environments. And the resources are there, but they are not used adequately due to lack of execution". (**PSSI**: 1, 4). |
| D F | 4-9 LOW 10-15MEDIUM 16-20 HIGH | 12,9 | 4,1 | "A lot of fragmentation within the institutions and a lack of capacity for integrality in the work at the territorial level" (**PSSI**: 3). |
|  |  |  |  | "There is no institutional policy to follow up on the process of adherence to dengue guidelines, nor is it an institutional priority within its organizational processes" (**FGP**:10). |
| D G | 2-4 LOW 5-6 MEDIUM 7-10 HIGH | 5,9 | 2,1 | "A major limitation in terms of the capacity of the Ministry and also of the Institute to accompany, to advise, to monitor and control that a good job is being done at the level of the territories and in this time of pandemic, well, I would say that a good part of this work was practically abandoned" (**PSSI**: 3). |
|  |  |  |  | "Research institutions or academic institutions have a very important job to do in articulated work with the institutions, with the health authorities of the territorial entities and even among themselves; many times there is also competition among the same academic and research institutions or in channeling resources that also fragment the possibility of having an accompaniment that produces a better impact on the response that should be given to problems such as dengue" (**PSSI**: 3). |

* DA: Guideline determinants; DB: Individual health professional factors; DC: Patient factors; DD: Professional interactions factors;

DE: Incentive and resource factors; DF: Capacity for organizational change; DG: Social, political and legal factors. **Score range:

Minimum and maximum score for each domain stratified into categories; evaluated on a 5-point Likert scale 1=Strongly disagree,

2=Disagree, 3=Strongly agree, 4=Agree, 5=Strongly agree. *** Mean and SD: Standard Deviation.

**GACIPD**: Guía de Atención Integral del Dengue (Comprehensive Care Guide for Dengue)

**PSSI**: Participant in the semi-structured interview

**FGP**: Focus Group Participant.
